# Genomics of body fat percentage may contribute to sex bias in anorexia nervosa

**DOI:** 10.1002/ajmg.b.32709

**Published:** 2018-12-28

**Authors:** Christopher Hübel, Héléna A. Gaspar, Jonathan R. I. Coleman, Hilary Finucane, Kirstin L. Purves, Ken B. Hanscombe, Inga Prokopenko, MAGIC investigators, Mariaelisa Graff, Julius S. Ngwa, Tsegaselassie Workalemahu, Eating Disorders Working Group of the Psychiatric Genomics Consortium, Major Depressive Disorder Working Group of the Psychiatric Genomics Consortium, Schizophrenia Working Group of the Psychiatric Genomics Consortium, Tourette Syndrome/Obsessive‐Compulsive Disorder Working Group of the Psychiatric Genomics Consortium, Paul F. O'Reilly, Cynthia M. Bulik, Gerome Breen

**Affiliations:** ^1^ Social, Genetic & Developmental Psychiatry Centre Institute of Psychiatry, Psychology & Neuroscience, King's College London London United Kingdom; ^2^ UK National Institute for Health Research (NIHR) Biomedical Research Centre South London and Maudsley Hospital London United Kingdom; ^3^ Department of Medical Epidemiology and Biostatistics Karolinska Institutet Stockholm Sweden; ^4^ Schmidt Fellows Program Broad Institute of MIT and Harvard Cambridge Massachusetts; ^5^ Department of Medical and Molecular Genetics King's College London, Guy's Hospital London United Kingdom; ^6^ Section of Genomics of Common Disease, Department of Medicine Imperial College London London United Kingdom; ^7^ Department of Epidemiology University of North Carolina Chapel Hill North Carolina; ^8^ Department of Biostatistics Johns Hopkins Bloomberg School of Public Health Baltimore Maryland; ^9^ Department of Biostatistics Boston University School of Public Health Boston Massachusetts; ^10^ Epidemiology Branch, Division of Intramural Population Health Research Eunice Kennedy Shriver National Institute of Child Health and Human Development, National Institutes of Health Bethesda Maryland; ^11^ Department of Psychiatry University of North Carolina at Chapel Hill Chapel Hill North Carolina; ^12^ Department of Nutrition University of North Carolina at Chapel Hill Chapel Hill North Carolina

**Keywords:** eating disorder, fat‐free mass, female, genetic correlation, GWAS, shared genetics

## Abstract

Anorexia nervosa (AN) occurs nine times more often in females than in males. Although environmental factors likely play a role, the reasons for this imbalanced sex ratio remain unresolved. AN displays high genetic correlations with anthropometric and metabolic traits. Given sex differences in body composition, we investigated the possible metabolic underpinnings of female propensity for AN. We conducted sex‐specific GWAS in a healthy and medication‐free subsample of the UK Biobank (*n* = 155,961), identifying 77 genome‐wide significant loci associated with body fat percentage (BF%) and 174 with fat‐free mass (FFM). Partitioned heritability analysis showed an enrichment for central nervous tissue‐associated genes for BF%, which was more prominent in females than males. Genetic correlations of BF% and FFM with the largest GWAS of AN by the Psychiatric Genomics Consortium were estimated to explore shared genomics. The genetic correlations of BF%_male_ and BF%_female_ with AN differed significantly from each other (*p* < .0001, *δ* = −0.17), suggesting that the female preponderance in AN may, in part, be explained by sex‐specific anthropometric and metabolic genetic factors increasing liability to AN.

## 1 INTRODUCTION

Anorexia nervosa (AN) is one of the most lethal psychiatric disorders and has established environmental and genetic risk factors (Chesney, Goodwin, & Fazel, 2014; Keshaviah et al., 2014). Female sex is the most robust and replicated risk factor, with nine females affected for each male case observed (Bulik et al., 2006; Micali, Hagberg, Petersen, & Treasure, 2013; Steinhausen & Jensen, 2015). Although historic diagnostic criteria for AN may have favored detection in females (e.g., presence of the amenorrhea criterion), most schemata did allow for the diagnosis of AN in males (American Psychiatric Association, 2013; World Health Organization, 1992). The focus of most work on gender differences in AN has been on sociocultural factors, such as personal evaluation of physical appearance and social pressures to be thin (Bakalar, Shank, Vannucci, Radin, & Tanofsky‐Kraff, 2015) although models based on biological and hormonal factors, such as growth, sex, and appetite‐regulating hormone abnormalities have also been posited (Culbert, Racine, & Klump, 2016; Schorr & Miller, 2017). However, collectively findings to date are not yet able to account for the widely disparate prevalences by sex.

The marked alterations in body composition, including fat mass (FM), fat‐free mass (FFM), and bone mineral density observed in AN are clinical characteristics of the illness, but have generally been considered to be sequelae of starvation (Westmoreland, Krantz, & Mehler, 2016). Females with AN show significantly greater FM deficits than affected males (Nagata et al., 2017) and, even after recovery, some individuals do not restore healthy body fat percentages (BF%; El Ghoch, Calugi, Lamburghini, & Dalle Grave, 2014). Moreover, lower BF% is a major risk factor for relapse (Bodell & Mayer, 2011). The causes of these particular sex differences have not yet been fully investigated.

Both AN and body composition as measured by bioelectrical impedance analysis are heritable (Schousboe et al., 2004; Tarnoki et al., 2014; Table S1). Significant negative single nucleotide polymorphism‐based autosomal genetic correlations (*SNP‐r*_*g*_) between AN and body mass index (BMI) and BF% were observed by the largest GWAS of AN conducted by the Eating Disorders Working Group of the Psychiatric Genomics Consortium (PGC‐ED; Duncan et al., 2017; Watson et al., 2018). This suggests shared etiology between those anthropometric traits and AN. Furthermore, AN shares common genetic variation with metabolic traits, such as insulin sensitivity and cholesterol. This revealed, for the first time, that a component of the genetic risk for AN is related to body composition and metabolism (Duncan et al., 2017; Hinney et al., 2017).

Phenotypic sex differences in body composition are also present in the general population; discernible as early as adolescence, females have on average higher BF% (Flegal et al., 2009), and less visceral adipose tissue and FFM than males (Paus, Wong, Syme, & Pausova, 2017), partially due to differences in adipocyte metabolism (Cheung & Cheng, 2016; Karastergiou & Fried, 2017; Link & Reue, 2017). Moreover, epidemiological findings indicate a female predominance at both tails of BMI, in extreme obesity (Kelly, Yang, Chen, Reynolds, & He, 2008; Lovre & Mauvais‐Jarvis, 2015) and in AN (Steinhausen & Jensen, 2015). Recent evidence shows clear biological sex differences in metabolism in rodent models (Arnold, 2017) and in humans (Mauvais‐Jarvis, 2015).

The observed phenotypic sex differences in body composition across the lifespan are partially due to genetic factors (Table S1 and Figure S1; Silventoinen et al., 2016, 2017). Heritability estimates from twin studies (*twin‐h* ^2^) of these epidemiological sex differences unveiled that *twin‐h* ^2^ estimates of BMI—a proxy of BF%—vary across the lifespan and show sex‐specific patterns, most apparent at the age of 13 years, from 20 to 30, and between ages 70 and 80 (Table S1 and Figure S1; Silventoinen et al., 2016, 2017). Although the *twin‐h* ^2^ varies somewhat, the specific genetic factors influencing BMI remain stable from decade to decade postadolescence, whereas environmental effects appear to change across time, especially in females (Haberstick et al., 2010). Additionally, several GWAS of proxy measures of BF% (Heid et al., 2010; Lindgren et al., 2009; Pulit et al., 2018; Randall et al., 2013; Winkler et al., 2017) and of BF% itself (Kilpeläinen et al., 2011; Lu et al., 2016) show clear sex differences in genome‐wide significant genomic loci and documented female‐specific heterogeneity in the genomic architecture extensively (for review, see Link & Reue, 2017; Pulit, Karaderi, & Lindgren, 2017; Small et al., 2018). Furthermore, studies have shown that BMI GWAS show tissue‐specific enrichment for the central nervous system (CNS; Finucane et al., 2015, 2018), whereas waist‐to‐hip ratio adjusted for BMI GWAS showed enrichment for adipose tissue (Finucane et al., 2018).

Convergent epidemiological and genetic findings show that the regulation of body composition varies between the sexes and is substantially influenced by both genetic and environmental factors. The primary goal of this study is to investigate whether a sex‐specific analysis of genetic determinants of body composition may partially explain the observed female preponderance in AN. We utilize new GWAS summary statistics from the PGC‐ED with about 16,000 cases, capitalizing on the availability of detailed and highly standardized body composition measurements and genetic data of 155,961 healthy and medication‐free individuals in the UK Biobank. Together, these provide a unique opportunity for a powerful investigation of the sex specificity of the genetic underpinnings of body composition and psychiatric traits and their relationship with AN.

## 2 METHODS

### 2.1 Genome‐wide association study of AN by the Eating Disorders Working Group of the Psychiatric Genomics Consortium

The meta‐analysis of GWAS on AN was a combined effort by the AN Genetics Initiative (Kirk et al., 2017; Thornton et al., 2018) and the PGC‐ED (www.med.unc.edu/pgc) and comprised 33 cohorts from 17 countries (Table S3) with 16,992 AN cases and 55,525 controls (Watson et al., 2018). The GWAS included 72,358 females (16,531 of whom are cases) and 24,454 males (460 of whom are cases; Table S2). The analysis includes additional samples from the Genetic Consortium for AN, the Wellcome Trust Case Control Consortium 3 (Boraska et al., 2014), and the UK Biobank (Sudlow et al., 2015). Case definitions established a lifetime diagnosis of AN via hospital or register records, structured clinical interviews, or online questionnaires based on standardized criteria—DSM‐III‐R, DSM‐IV, ICD‐8, ICD‐9, or ICD‐10—(American Psychiatric Association, 2013; World Health Organization, 1992), whereas in the UK Biobank cases self‐reported a diagnosis of AN (Davis et al., 2018). Quality control, imputation, GWAS, and meta‐analysis followed the standardized pipeline of the PGC, Rapid Imputation Consortium Pipeline (Ricopili; https://github.com/Nealelab/ricopili/tree/master/rp_bin). SNPs were excluded if they had a minor allele frequency (MAF) smaller than 1%, if no call was made in more than 2% of samples following imputation, if they were imputed with low confidence (INFO<0.7), or if they deviated substantially from Hardy–Weinberg equilibrium (controls *p* < 10^−6^, cases *p* < 10^−10^). Individuals were excluded if they showed inbreeding coefficients >0.2, or evidence of DNA contamination. Ancestry outliers were removed based on plotting of the first two principal components (PCs). The analysis was performed using imputed variant dosages and an additive model. The SNP‐based heritability (*SNP‐h* ^2^) of AN calculated using these data was 17% (*SE* = 1%), suggesting that a substantial fraction of the heritability of AN stems from common genetic variation across all autosomes (Watson et al., 2018)**.**

### 2.2 GWASs of body composition: Study design and participants

Our study includes a cross‐sectional analysis of the baseline data from the epidemiological resource UK Biobank (www.ukbiobank.ac.uk; Allen, Sudlow, Peakman, Collins, & UK Biobank, 2014; Sudlow et al., 2015). To identify genetic variation associations with BF% and FFM that are not confounded by illnesses and their downstream effects or metabolism‐changing medication, we applied stringent exclusion criteria (Table S2). Due to this trait‐specific medication and illness filtering, the final analysis included 155,961 (45% female) healthy and drug‐free European ancestry participants comprising 32% of the genotyped UK Biobank participants. European ancestry was defined by 4‐means clustering of the first two PCs from the genetic data (Warren et al., 2017). Phenotypic characteristics separated by sex are presented in Table 1. All statistics were calculated in R 3.4.1 if not otherwise stated.

**Table 1:** Phenotypic characteristics of individuals in the analyses

	Meta‐analyzed	Female	Male
Number (%)	155,961	70,700 (45%)	85,261 (55%)
Age (years)	54.9 ± 8.1	54.8 ± 8.0	55.0 ± 8.2
Height (cm)	170.4 ± 9.3	163.0 ± 6.2	176.4 ± 6.7
Weight (kg)	78.1 ± 15.1	69.6 ± 12.6	85.1 ± 13.2
BMI (kg/m^2^)	27.0 ± 4.2	26.2 ± 4.6	27.4 ± 3.8
Waist circumference (cm)	89.4 ± 12.6	82.3 ± 11.3	95.3 ± 10.3
Hip circumference (cm)	102.5 ± 8.1	102.0 ± 9.3	103.0 ± 6.9
Waist‐to‐hip ratio	0.9 ± 0.1	0.8 ± 0.1	0.9 ± 0.1
Body fat (%)	29.3 ± 8.2	35.3 ± 6.7	24.4 ± 5.5
Fat mass (kg)	23.0 ± 8.5	25.3 ± 9.1	21.2 ± 7.5
FFM (kg)	55.1 ± 11.6	44.4 ± 4.6	63.9 ± 7.4
SES, Townsend deprivation index	−1.6 ± 2.9	−1.7 ± 2.8	−1.7 ± 2.9

BMI = body mass index; FFM = fat‐free mass; SES = socioeconomic status.

Data are *n* (%), or mean (*SD*).

### 2.3 Body composition assessment in healthy participants

Body composition was assessed with a rigorous and highly standardized protocol by UK Biobank using the same Tanita BC‐418 MA machines (Tanita Corporation, Arlington Heights, IL) for every participant. This body composition analyzer calculates FFM and FM from raw bioelectrical impedance data, using standard formulas including sex, age, height, and athleticism. Individuals whose hydration status might be compromised (e.g., suffering from diabetes mellitus or other endocrine diseases) were excluded (Table S3). Bioelectrical impedance technology has been extensively validated (Genton et al., 2003; Kyle et al., 2004; Lu et al., 2016), and results in more reliable estimates of body adiposity than BMI for healthy individuals (Mazzoccoli, 2016; Tanamas et al., 2016). Therefore, bioelectrical impedance analysis is the most feasible method in very large epidemiological samples, such as the UK Biobank, compared with proxy measures of adiposity, and does not expose participants to radiation unlike dual‐energy X‐ray absorptiometry.

### 2.4 GWASs on body composition

We calculated sex‐specific GWAS on residualized BF% and FFM, using BGENIE v1.2 (Bycroft et al., 2018). Our final analyses included 7,794,483 SNPs and insertion–deletion variants with an MAF >1%, imputation quality scores >0.8, and that were genotyped, or present in the Haplotype Reference Consortium (HRC) reference panel used for imputation by UK Biobank (McCarthy et al., 2016). We used an additive model on the imputed dosage data provided by UK Biobank, and residualized phenotypes prior to GWAS for factors related to assessment center, genotyping batch, smoking status, alcohol consumption, menopause, and for continuous measures of age, and socioeconomic status (SES) measured by the Townsend deprivation index (Townsend, 1987) as independent variables. We accounted for underlying population stratification by also including the first six PCs, calculated on the genotypes of the European subsample. We then meta‐analyzed these sex‐specific GWAS using METAL (http://csg.sph.umich.edu/abecasis/metal/; Willer, Li, & Abecasis, 2010) using an inverse variance weighted model with a fixed effect, to obtain sex‐combined results. Significantly associated SNPs (*p* < 5 × 10^−8^) were considered as potential index SNPs. SNPs in LD (*r* ^2^ > 0.2) with a more strongly associated SNP within 3,000 kb were assigned to the same locus using Functional Mapping and Annotation (FUMA; Watanabe, Taskesen, van Bochoven, & Posthuma, 2017). Overlapping clumps additionally were merged with a second clumping procedure in FUMA merging all lead SNPs with *r* ^2^ = 1 to genomic loci. After clumping, independent genome‐wide significant loci (5 × 10^−8^) were compared with entries in the NHGRI‐EBI GWAS catalog (MacArthur et al., 2017) using FUMA (Watanabe et al., 2017). Sex‐specific loci are defined as reaching genome‐wide significance (5 × 10^−8^) in either females or males while not showing at least suggestive significance in the opposite sex (5 × 10^−6^) with differences in beta estimates that remain significant after Bonferroni correction for the total number of significant genomic loci.

### 2.5 Genome‐wide SNP‐based heritability and partitioned heritability

Using BOLT‐LMM (Loh et al., 2015) on genotyped, genome‐wide, common genetic variants and linkage disequilibrium score regression (LDSC) implemented in LDSC v.1.0.0 (Bulik‐Sullivan et al., 2015) on genome‐wide summary statistics, we calculated the total phenotypic variance explained by common autosomal SNPs, SNP‐based heritability (*SNP‐h* ^2^). We included all genotyped and imputed autosomal variants for BF% and FFM and used the LD score reference files provided with the software. We tested for differences between the heritabilities by calculating *SE* using a block jackknife method implemented into the software. To identify tissue types associated with BF% and FFM, we performed a partitioned heritability analysis in LDSC v.1.0.0, ranking 10 cell type groups based on contribution to heritability after controlling for the effects of 53 functional annotations (Finucane et al., 2015).

### 2.6 Genetic correlations

Using an analytic extension of LDSC (Bulik‐Sullivan et al., 2015), we calculated SNP‐based bivariate genetic correlations (*SNP‐r*_*g*_) across the autosomes to examine the genetic overlap between AN and metabolic and psychiatric GWAS summary statistics. First, we calculated *SNP‐r*_*g*_s between anthropometric traits, namely our BF% and FFM GWASs with GWASs of childhood BMI (~8 years; Felix et al., 2016), childhood obesity (Bradfield et al., 2012), childhood FFM (Medina‐Gomez et al., 2017), adult FFM (Zillikens et al., 2017), and adolescence and young adulthood BMI (~15–25 years; Graff et al., 2013), to estimate the genomic overlap of body composition between different periods of life. Second, we calculated *SNP‐r*_*g*_s of these anthropometric traits across the lifespan with AN.

Additionally, we computed *SNP‐r*_*g*_s of AN (Supporting Information) with glycemic traits, such as insulin sensitivity assessed by the insulin resistance homeostatic model assessment (HOMA‐IR), fasting glucose, and insulin concentrations (Lagou, Mägi, & Hottenga, 2018; Manning et al., 2012; Scott et al., 2012), to investigate potential mediation of the relationship between body fat and AN. Physical activity is reported to be increased in AN patients (Achamrah, Coëffier, & Déchelotte, 2016; Shroff et al., 2006); therefore, we estimated the genetic overlap between physical activity (Hanscombe, 2018, Unpublished, Supporting Information) and AN. We explored the genomic contribution to the comorbidity of AN with psychiatric disorders and traits, including major depressive disorder (MDD; Major Depressive Disorder Working Group of the Psychiatric GWAS Consortium et al., 2013), anxiety (Purves et al., 2017), schizophrenia (Schizophrenia Working Group of the Psychiatric Genomics Consortium et al., 2014), obsessive–compulsive disorder (OCD; International Obsessive Compulsive Disorder Foundation Genetics Collaborative (IOCDF‐GC) and OCD Collaborative Genetics Association Studies (OCGAS), 2018), and neuroticism (Coleman, 2017, Unpublished, Supporting Information), as well as educational attainment (Okbay et al., 2016) by calculating *SNP‐r*_*g*_s. Information on all GWAS is presented in Table S4.

### 2.7 Sex‐specific analyses of genomic determinants

We investigated differences between sexes in heritability and genetic architecture to identify sex‐specific liability driven by genomic factors. We examined differences (*δ*) in the *SNP‐h* ^2^ estimates between males and females using a block jackknife approach (Supporting Information) and tested whether the *SNP‐r*_*g*_s between females and males were different from 1 to identify potential genetic differences related to sex. We calculated the *SNP‐r*_*g*_ of the female and male GWASs with AN separately to investigate the differences in the relationship of these sex differences with the risk for AN. To test the statistical significance of all estimates, we calculated their *SE* and corresponding *p* value by applying a block jackknife method, as described and implemented in LDSC v1.0.0 by Bulik‐Sullivan et al. (2015) and in our Supporting Information.

As a sensitivity analysis, we repeated all *SNP‐r*_*g*_ analysis with a female‐only GWAS of AN. However, due to the small number of male AN cases, it was impossible to perform a male‐only analysis. All methods are described in more detail in the Supporting Information. Stringent multiple testing correction was performed on each analysis, using matrix decomposition to detect the effective number of tests and subsequent Bonferroni correction of the *p* value thresholds.

## 3 RESULTS

### 3.1 GWAS of AN

The AN GWAS resulted in eight genome‐wide significant loci and showed enrichment for CNS cell types. It genetically correlated with a broad range of metabolic and psychiatric phenotypes, mirroring clinically observed comorbidity (for details, see Duncan et al., 2017 ; Watson et al., 2018).

### 3.2 GWAS of body composition measures in the UK Biobank

**Figure 1:**
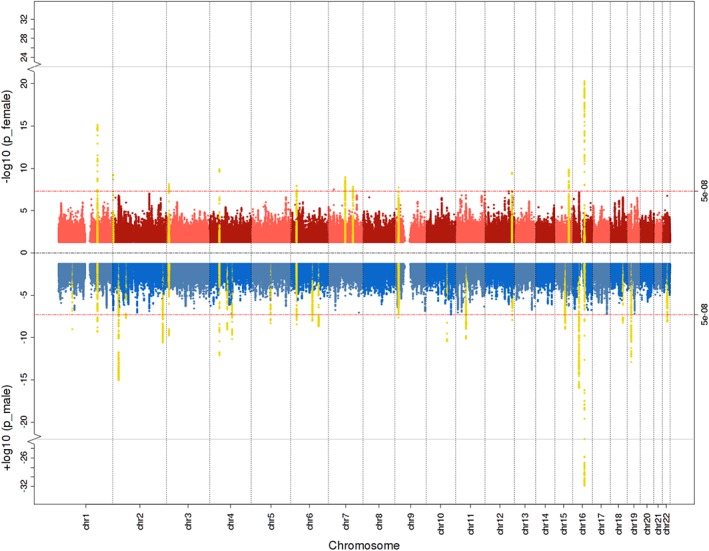
Miami plot for female (red), male (blue), and meta‐analyzed (yellow) genome‐wide body fat percentage (BF%) associations. Significant loci from the sex‐combined analyses are highlighted in yellow if they also reached genome‐wide significance in the sex‐specific genome‐wide association studies (GWASs). The genome‐wide significance threshold *p* < 5 × 10^−8^ is represented by the red horizontal lines. Chr = chromosome

**Figure 2:**
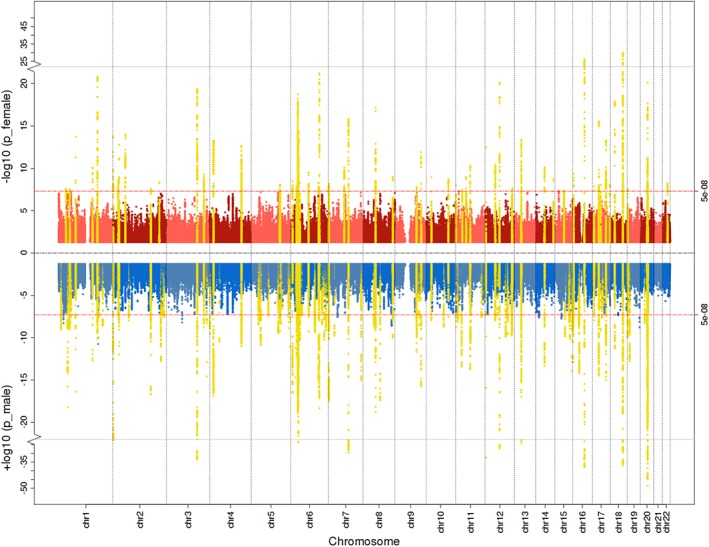
Miami plot for female (red), male (blue), and meta‐analyzed (yellow) genome‐wide fat‐free mass (FFM) associations. Significant loci from the sex‐combined analyses are highlighted in yellow if they also reached genome‐wide significance in the sex‐specific genome‐wide association studies (GWASs). The genome‐wide significance threshold *p* < 5 × 10^−8^ is represented by the red horizontal lines. Chr = chromosome

After quality control, we performed sex‐stratified association analyses on the continuous outcomes of BF% and FFM. Minimal inflation due to population stratification or other systematic biases was indicated by LDSC intercepts between 1.02 and 1.10 and lambda median statistic inflation values (λ_median_) between 1.18 and 1.59 (Table S4 and Figure S3a,b). We identified 34 independent loci associated with meta‐analyzed BF% that are not reported to be associated with anthropometric traits in the GWAS catalog (MacArthur et al., 2017) and replicated 42 independent genome‐wide significant results (*p* < 5 × 10^−8^) after LD‐based and distance‐based clumping (Figure 1, Figure S4a, Table S5a,b). We identified one male‐specific locus in BF% (Table S5a). The meta‐analyzed GWAS of FFM yielded 83 novel loci and replicated 78 genomic risk loci previously associated with anthropometric traits (Figure 2, Figure S3b, Table S6a,b). We identified 13 male‐specific genomic loci in FFM (Table S6a). All genomic regions, region plots thereof, their annotations, including nearby protein coding genes (within 100 kb), and previous entries in the GWAS catalog are published on FUMA (http://fuma.ctglab.nl/browse) entries 20–25. Summary statistics are available for download www.topherhuebel.com/GWAS.

### 3.3 Genome‐wide SNP‐based and partitioned heritability

**Figure 3:**
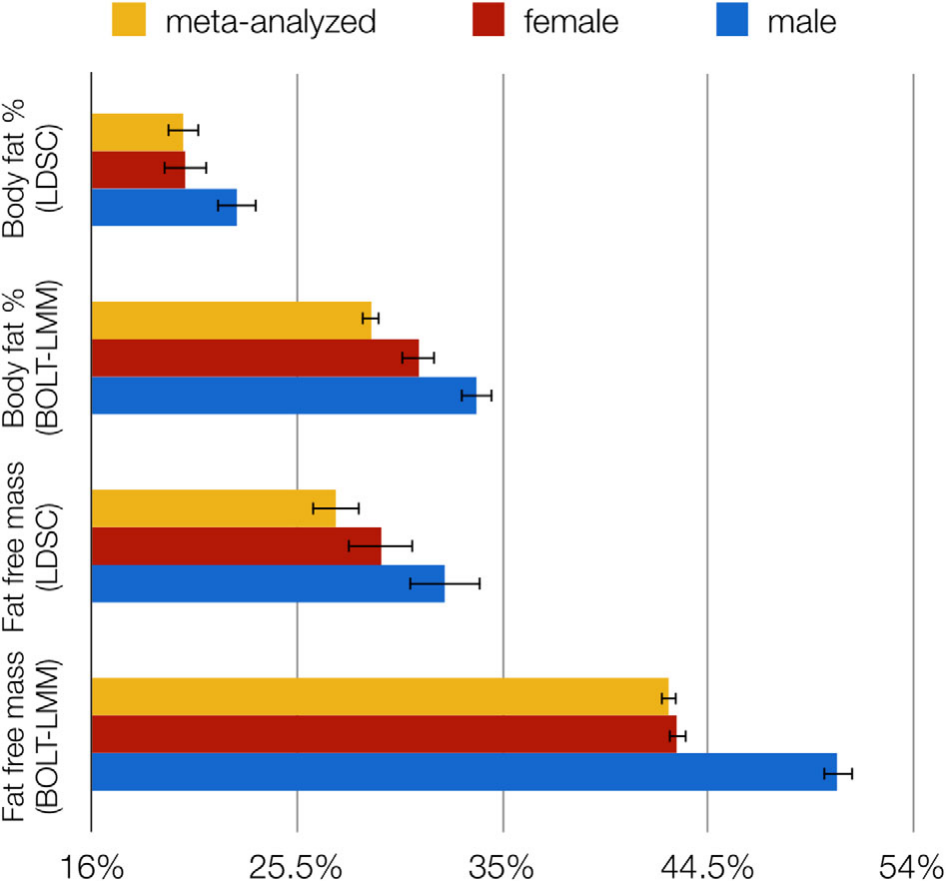
Sex‐specific single nucleotide polymorphism‐based heritability estimates (*SNP‐h* ^2^) for body fat percentage and fat‐free mass calculated by BOLT‐LMM (Loh et al., 2015) and linkage disequilibrium score regression (LDSC; Bulik‐Sullivan et al., 2015). Error bars represent *SE*. All estimated *SNP‐h* ^2^ were statistically significant

The *SNP‐h* ^2^ for BF% ranged between 29 and 33%, and for FFM between 43 and 51% (Figure 3), while that for AN is about 17–20% with an assumed population prevalence of 0.9% (Duncan et al., 2017; Watson et al., 2018). The *SNP‐h* ^2^ of FFM_male_ measured by LDSC was significantly higher than the *SNP‐h* ^2^ of FFM_meta_ (*p* < .001, *δSNP‐h* ^2^ = 5.6%). However, neither the *SNP‐h* ^2^ estimates for BF% nor for FFM measured by LDSC differed significantly between the sexes.

Partitioned heritabilities can estimate the proportion of the overall *SNP‐h* ^2^ that can be attributed to different cell type groups. BF%_female_ showed an significant enrichment for the CNS cell type group with 14% of SNPs explaining an estimated 40% of the *SNP‐h* ^2^ (*p* = .004), whereas BF%_male_ was significantly enriched for the “other” cell type group that contains adipose tissue with 20% of SNPs explaining an estimated 57% of the *SNP‐h* ^2^ (*p* = .004; Figure S4a,b). The FFM_female_ and FFM_male_ were enriched for connective and bone tissue with 11% of SNPs explaining an estimated 47% of *SNP‐h* ^2^ in both sexes (*p*_female_ = 6.65 × 10^−6^; *p*_male_ = 2.29 × 10^−7^; Figure S5a,b). The meta‐analyzed FFM_both_ was also enriched for skeletal muscle with 10% of SNPs explaining an estimated 37% of *SNP‐h* ^2^ (*p* = .004, Figure S5c).

### 3.4 Genetic correlations of anthropometric traits across the lifespan

The significant *SNP‐r*_*g*_ between BF%_meta_ and BMI_childhood_ was 0.46 (*SE* = 0.04; *p* = 6.11 × 10^−32^) and between BF%_meta_ and BMI_adolescence/young adulthood_ was 0.48 (*SE* = 0.05; *p* = 9.24 × 10^−25^). Similarly, FFM_childhood_ and FFM_adulthood_ showed a significant *SNP‐r*_*g*_ of 0.69 (*SE* = 0.10; *p* = 2.70 × 10^−12^) and FFM_childhood_ also correlated genetically with FFM_meta_ in our UK Biobank sample (*SNP‐r*_*g*_ *=* 0.30; *SE* = 0.04; *p* = 3.24 × 10^−12^).

**Figure 4:**
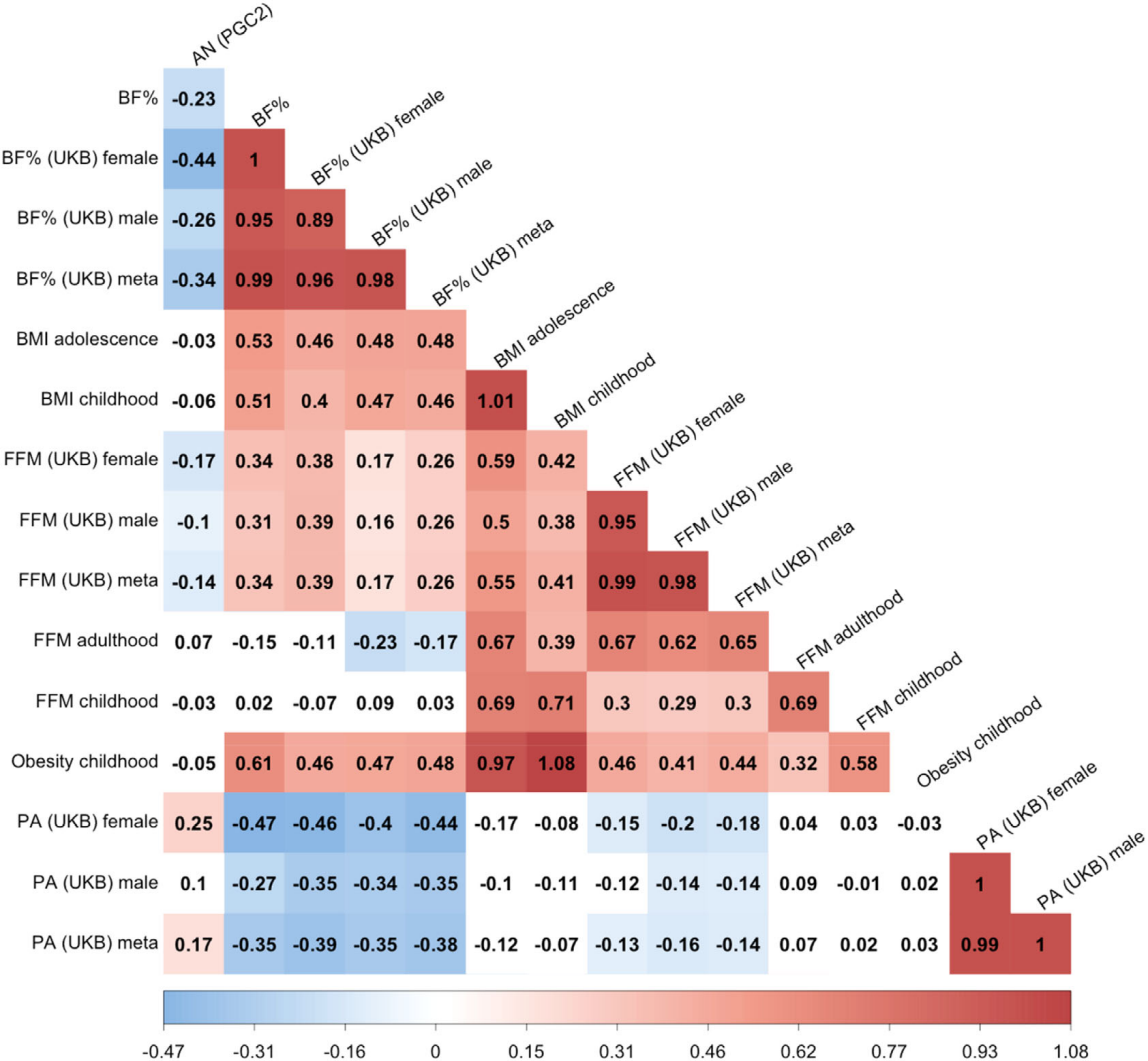
Heatmap of sex‐specific bivariate single nucleotide polymorphism‐based genetic correlations (*SNP*‐r^2^_g_) of body fat percentage, BMI, fat‐free mass, physical activity, and obesity with AN. The strength of the correlation is reflected in the hue. Blue colors are negative *SNP‐r*_*g*_s, meaning that the same genetic variants influence both traits in opposite directions, and red are positive *SNP‐r*_*g*_s meaning that the same genetic variants influence traits in the same direction. Colored squares are significant after correction for multiple comparisons by matrix decomposition and Bonferroni correction (*p*_Bonferroni_ = .05/10). The *SNP‐r*_*g*_s were calculated by linkage disequilibrium score regression (LDSC). AN = anorexia nervosa; BF% = body fat percentage; BMI = body mass index; FFM = fat‐free mass; PA = physical activity; PGC2 = 2nd freeze psychiatric genomics consortium; UKB = UK Biobank

BF%_meta_ and FFM_meta_ correlated genetically (*SNP‐r*_*g*_ = 0.26; *SE* = 0.02; *p* = 3.95 × 10^−26^). The *SNP‐r*_*g*_ between BF%_female_ and BF%_male_ was significantly less than 1 (*SNP‐r*_*g*_ = 0.89, *SE* = 0.03; *p*_*=1*_ = .0005), indicating heterogeneity in the genomic architecture between females and males (Figure 4).

### 3.5 Sex‐specific genetic correlations with AN

We calculated *SNP‐r*_*g*_ between the sex‐specific and sex‐combined GWAS with AN to investigate sex differences. The genetic correlation between BF%_female_ and AN was −0.44 (*SE* = 0.04; *p* = 8.28 × 10^−27^), whereas that between BF%_male_ and AN was −0.26 (*SE* = 0.04; *p* = 1.04 × 10^−13^). These *SNP‐r*_*g*_ were significantly different from each other (*δSNP‐r*_*g*_ = −0.17; *SE* = 0.04; *p* = 4.23 × 10^−5^). AN showed a significant genetic correlation with FFM_meta_ (*SNP‐r*_*g*_ = −0.14; *SE* = 0.03; *p* = 5.79 × 10^−6^) Physical activity_female_ showed a significant *SNP‐r*_*g*_ with AN (*SNP‐r*_*g*_ = 0.25; *SE* = 0.06; *p* = 1.10 × 10^−5^), but physical activity_males_ did not (*SNP‐r*_*g*_ = 0.10; *SE* = 0.06; *p* = .07). However, this difference was not statistically significant (*δSNP‐r*_*g*_ = −0.13; *SE* = 0.07; *p* = .05; Figure 4) after multiple testing correction.

**Figure 5:**
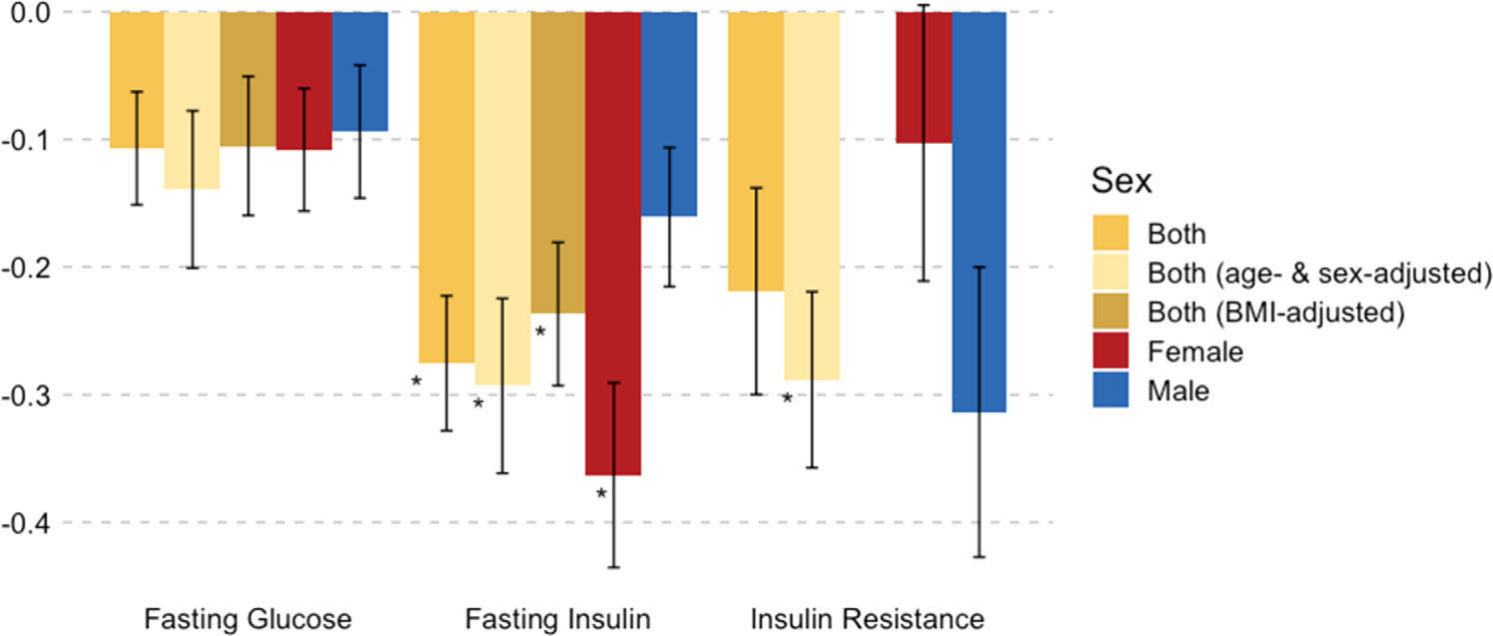
Sex‐specific bivariate single nucleotide polymorphism‐based genetic correlations (*SNP‐r*_*g*_) of fasting glucose, fasting insulin, and insulin resistance assessed by the HOMA‐IR with AN. The *SNP‐r*_*g*_s were calculated by linkage disequilibrium score regression (LDSC). Significant *SNP‐r*_*g*_s are marked with an asterisk (*) after correction for multiple comparisons by matrix decomposition and Bonferroni correction (*p*_Bonferroni_ = .05/28). The error bars depict the *SE*. Summary statistics for BMI‐adjusted HOMA‐IR were not available. AN = anorexia nervosa; BMI = body mass index; HOMA‐IR = insulin resistance by homeostatic model assessment

BMI‐adjusted fasting insulin concentrations and AN were genetically correlated (*SNP‐r*_*g*_ = −0.24; *SE* = 0.06; *p* = 2.31 × 10^−5^). Fasting insulin_female_ was genetically correlated with AN (*SNP‐r*_*g*_ = −0.36; *SE* = 0.07; *p* = 5.29 × 10^−7^), but not fasting insulin_male_ (*SNP‐r*_*g*_ = −0.16; *SE* = 0.05; *p* = .003). However, this difference in *SNP‐r*_*g*_ between sexes did not reach significance (*δSNP‐r*_*g*_ = −0.19; *SE* = 0.08; *p* = .02) after multiple testing correction. Sex‐ and age‐adjusted insulin resistance (HOMA‐IR) correlated significantly with AN (*SNP‐r*_*g*_ = −0.29, *SE* = 0.07; *p* = 2.83 × 10^−5^; Figure 5), but no sex differences were observed.

**Figure 6:**
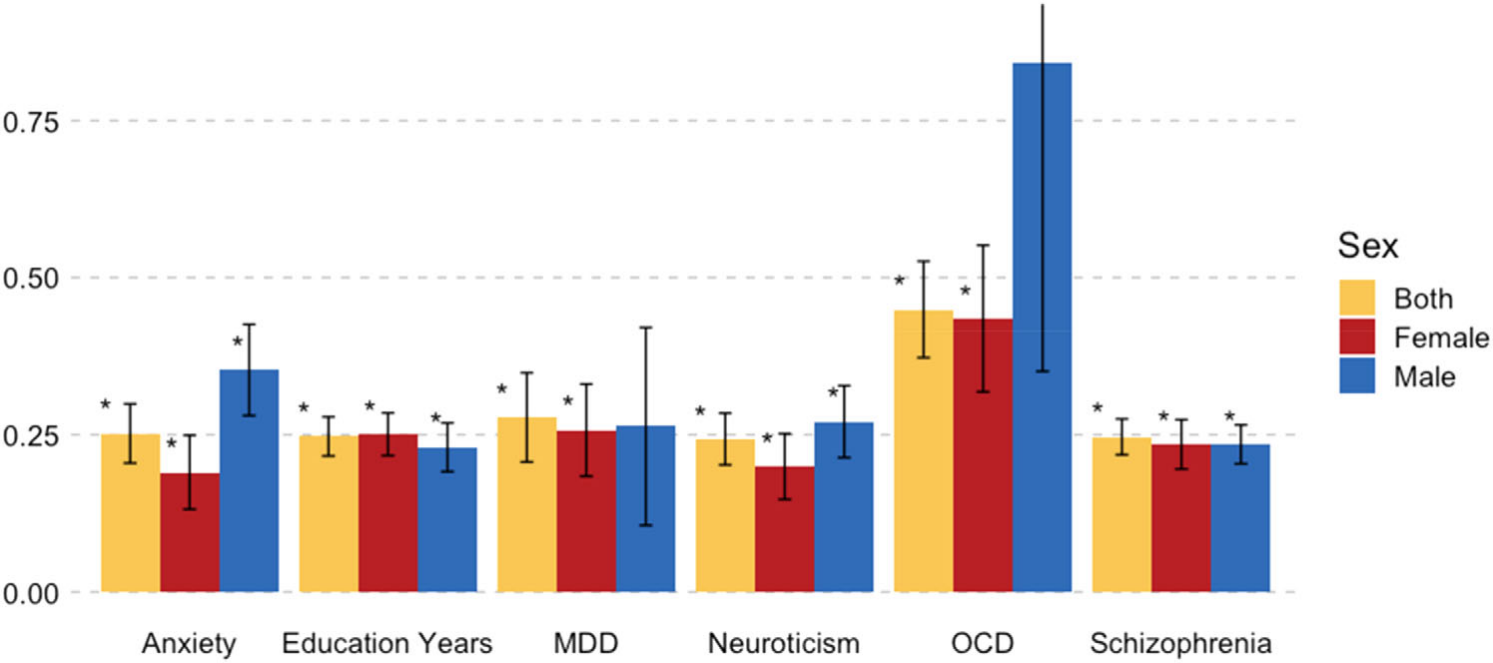
Sex‐specific bivariate single nucleotide polymorphism‐based genetic correlations (*SNP‐r*_*g*_) of probable anxiety disorder (anxiety), education years, MDD, neuroticism, OCD, and schizophrenia with anorexia nervosa. The *SNP‐r*_*g*_s were calculated by linkage disequilibrium score regression (LDSC). Significant *SNP‐r*_*g*_s are marked with an asterisk (*) after correction for multiple comparisons by matrix decomposition and Bonferroni correction (*p*_Bonferroni_ = .05/28). The error bars depict the *SE*. The *SE* of the OCD_male_ reaches above 1 and has been cut off. MDD = major depressive disorder; OCD = obsessive–compulsive disorder

AN was significantly correlated with MDD_female_ (*SNP‐r*_*g*_ = 0.26; *SE* = 0.07; *p* = 4.00 × 10^−4^) and anxiety_meta_ (*SNP‐r*_*g*_ = 0.25; *SE* = 0.05; *p* = 8.90 × 10^−8^). However, the difference between the male and female *SNP‐r*_*g*_ with AN was not significant in MDD (*δSNP‐r*_*g*_ = −0.004; *SE* = 0.16; *p* = .98). While the *SNP‐r*_*g*_ between education years in females and males was significantly different from 1 (*SNP‐r*_*g*_ = 0.91, *SE* = 0.02; *p* = 7.99 × 10^−5^), indicating sex differences, the *SNP‐r*_*g*_ of education years with AN did not differ between females and males (*δSNP‐r*_*g*_ = −0.02; *SE* = 0.03; *p* = .59; Figure 6). As sensitivity analysis, all *SNP‐r*_*g*_s were also calculated with a female only AN GWAS showing no meaningful differences (Table S8a).

## 4 DISCUSSION

The latest GWAS on AN by the PGC‐ED presented evidence for a reconceptualization of AN as a metabo‐psychiatric disorder by identifying significant *SNP‐r*_*g*_s of AN with a variety of metabolic phenotypes, including body composition, lipid metabolism, and glycemic traits (Duncan et al., 2017; Watson et al., 2018). We extended the findings on the relationship between BF% and AN by replicating that genomic effects on BF% differ by sex (Heid et al., 2010; Lindgren et al., 2009; Pulit et al., 2018; Randall et al., 2013; Winkler et al., 2017) and showing that female‐specific effects on BF% have a significantly greater genetic correlation with AN (*SNP‐r*_*g*_ = −0.44; *SE* = 0.04; *p* = 8.28 × 10^−27^) than male‐specific effects on BF% (*SNP‐r*_*g*_ = −0.26; *SE* = 0.04; *p* = 1.04 × 10^−13^). This suggests that a specific set of genomic variation may be differentially active in females and may increase the liability for AN. The partitioned heritability analyses of *SNP‐h* ^2^ showed that BF%_female_ was significantly enriched for CNS tissue while BF%_male_ was enriched for adipose tissue, recapitulating prior findings in sex‐combined samples (Finucane et al., 2015, 2018; Willer et al., 2009). This indicates a sex‐specific enrichment for BF% and that BF% has associated genetic variation underlying its biology thereby validating the use of bioelectrical impedance analysis to measure body compartments. Moreover, our findings suggest that different tissues may be implicated in the regulation of BF% in females and males.

In our analysis of body composition across the lifespan, BF%_childhood_, BF%_adolescence and young adulthood_, and FFM_childhood_ were not genetically correlated with AN, whereas BF%_adult_ and FFM_adult_ was. However, GWASs of BF% and BMI as well as FFM were well correlated across the lifespan with *SNP‐r*_*g*_ *s* of about ~0.60 across childhood, adolescence, young adulthood, and adulthood (Figure 4). This suggests that a proportion of BF%‐associated genomic variation may become operative at a later age and that this component may be correlated with risk for AN. This seems to overlap with the period—between 20 and 30 years of age—in which females and males show a significant difference in the *twin‐h* ^2^ of BMI (Figure S1; Silventoinen et al., 2016, 2017).

Additionally, we estimated *SNP‐r*_*g*_ of AN with sex‐specific GWASs of physical activity and glycemic traits to investigate potential moderators and mediators of the relationship between body fat and AN. Only physical activity_female_ and fasting insulin_female_ were significantly genetically associated with AN. However, the differences between female and male *SNP‐r*_g_s were only nominally significant for both traits and did not survive correction for multiple testing emphasizing the need for larger sample sizes to examine sex differences.

In our sex‐specific investigation of the contribution of psychiatric disorders and behavioral traits to AN, genomic variation associated with MDD in females and OCD in males suggested a possible sex effect in their *SNP‐r*_*g*_ with AN, but statistical tests did not confirm this. Power may be an issue; in particular, the current sample size of the OCD GWAS is relatively small. Consequently, some of our findings need to be interpreted cautiously, and this analysis should be repeated after much larger GWASs are available preferably with >10,000 cases of each sex. Some GWASs, however, are well powered and although the *SNP‐r*_*g*_ of education years between males and females was significantly lower than 1—similar to BF%—we did not observe sex differences in the *SNP‐r*_*g*_ of education years with AN, suggesting that metabolic traits may be more likely to contribute to the sex‐specific liability to AN than psychiatric or behavioral phenotypes.

Our investigation was limited by the small proportion of male AN cases in the primary AN GWAS (Table S2) not allowing for male‐only analyses. However, female‐only analyses did not show meaningful differences to the sex‐combined analyses (Table S8a). We were unable to include the X chromosome in the investigations as the genotype or summary level data for several GWASs in the PGC AN GWAS meta‐analysis were not available to us when the analyses were conducted. However, this should be incorporated in future studies. Most importantly, compared with prior BMI GWAS, our study benefited from arguably more homogeneously assessed body composition phenotypes, allowing us to differentiate between BF% and FFM more effectively (Kilpeläinen et al., 2011; Lu et al., 2016). Moreover, we adjusted for smoking behavior, alcohol consumption, and menopause and excluded participants taking weight altering medications and participants with somatic diseases or psychiatric disorders that affect body composition, such as cancers, diabetes, and MDD. This is a unique and important feature of our investigation and substantially reduced possible confounding of our GWAS.

### Conclusion

Our results add further evidence that AN is both a psychiatric and metabolic disorder and suggest that an age‐dependent specific set of genomic variation may be differentially active in females that influences body composition, which may also contribute to liability for AN. Our work could have therapeutic implications, by considering exploring approaches to using body composition measures or genetic markers of body composition as predictors of clinical course or adverse outcome, and as a component of personalized treatment that considers an individual's propensity to lose therapeutically restored weight. Some individuals may be at greater risk of relapse, for example, when confronted with periods of negative energy balance, and this could be addressed in personalized treatment and relapse prevention (Bulik, 2016). Sex‐specific genetic and biological factors may partially underlie increased risk for AN in females which suggests that new and focused studies of body composition and metabolism in AN patients could increase our understanding of AN etiology and response to treatment.

## Supporting information


**Figure S1** Twin‐based heritabilities (*twin‐h* ^2^) of body mass index (BMI) across the lifespan as calculated by ACE models from Silventoinen et al. Blue values represent males and red females with error bars depicting 95% confidence intervals. After the age of 19, heritabilities are represented for a whole decade (Silventoinen et al., 2016, 2017)
**Figure S2.** Analysis workflow chart. AN = anorexia nervosa; BMI = body mass index; GWAS = genome‐wide association study; LDSC = linkage score disequilibrium regression; MAGIC = Meta‐Analyses of Glucose and Insulin‐related traits Consortium; MDD = major depressive disorder; OCD = obsessive–compulsive disorder; PGC = Psychiatric Genomics Consortium; SSGAC = Social Science Genetic Association Consortium; UKB = UK Biobank
**Figure S3** (a) QQ plot for the body fat percentage (BF%) genome‐wide‐association study (GWAS). (b) QQ plot for the fat‐free mass (FFM) GWAS
**Figure S4** (a) Manhattan plot of the meta‐analyzed genome‐wide association study (GWAS) of body fat percentage (BF%). The red line represents the genome‐wide significance threshold of 5 × 10^−8^. Chr = chromosome. (b) Manhattan plot of the meta‐analyzed genome‐wide association study (GWAS) of fat‐free mass (FFM). The red line represents the genome‐wide significance threshold of 5 × 10^−8^. Chr = chromosome
**Figure S5** (a) Partitioned heritability by 10 cell type groups for body fat percentage in females. The black dashed lines at −log_10_(*P*) = 2.3 is the cutoff for Bonferroni significance. CNS = central nervous system SNP = single nucleotide polymorphism. (b) Partitioned heritability by 10 cell type groups for body fat percentage in males. The black dashed lines at −log_10_(*P*) = 2.3 is the cutoff for Bonferroni significance. CNS = central nervous system, SNP = single nucleotide polymorphism. (c) Partitioned heritability by 10 cell type groups for body fat percentage in the meta‐analyzed GWAS. The black dashed lines at −log_10_(*P*) = 2.3 is the cutoff for Bonferroni significance. CNS = central nervous system; SNP = single nucleotide polymorphism
**Figure S6** (a) Partitioned heritability by 10 cell type groups for fat‐free mass (FFM) in females. The black dashed lines at −log_10_(*P*) = 2.3 is the cutoff for Bonferroni significance. CNS = central nervous system, SNP = single nucleotide polymorphism. (b) Partitioned heritability by 10 cell type groups for FFM in males. The black dashed lines at −log_10_(*P*) = 2.3 is the cutoff for Bonferroni significance. CNS = central nervous system, SNP = single nucleotide polymorphism. (c) Partitioned heritability by 10 cell type groups for fat‐free mass (FFM) in the meta‐analyzed GWAS. The black dashed lines at −log_10_(*P*) = 2.3 is the cutoff for Bonferroni significance. CNS = central nervous system; SNP = single nucleotide polymorphism

**Table S1** Twin‐based (*twin‐h* ^2^) and single nucleotide polymorphism‐based heritability (*SNP‐h* ^2^) estimates derived from genome‐wide association studies (GWAS) for anorexia nervosa (AN) and anthropometric traits measured by bioelectrical impedance analysis of fat‐free mass (FFM), and body fat percentage (BF%)
**Table S2**. Sex data for the anorexia nervosa (AN) genome‐wide association study (GWAS) datasets
**Table S3**. Exclusion criteria by International Statistical Classification of Diseases (ICD‐10), British National Formulary (BNF), and UK Biobank variable
**Table S4**. Inflation statistics and heritability estimates on the observed scale of the genome‐wide association studies (GWAS). Attenuation ratio as calculated by Loh et al. (2017)
**Table S5** (a) Genome‐wide significant loci of the body fat percentage genome‐wide association study (GWAS) including heterogeneity measures of the meta‐analysis and a z‐test to test for significant differences between the sexes. Sex‐specific loci are defined as reaching genome‐wide significance (5 × 10^−8^) in either females or males, not reaching suggestive significance in the opposite sex (5 × 10^−6^) and the differences in beta estimates is significant after Bonferroni correction for the total number of significant genomic loci. (b) Genomic loci associated with anthropometric traits in published genome‐wide association studies (GWAS). Previous associations have been retrieved from the GWAS catalog (https://www.ebi.ac.uk/gwas/). Data extracted from Functional Mapping and Annotation of Genome‐Wide Association Studies (FUMA, http://fuma.ctglab.nl/)
**Table S6**. (a) Genome‐wide significant loci of the fat‐free mass genome‐wide association study (GWAS) including heterogeneity measures of the meta‐analysis and a z‐test to test for significant differences between the sexes. Sex‐specific loci are defined as reaching genome‐wide significance (5 × 10^−8^) in either females or males, not reaching suggestive significance in the opposite sex (5 × 10^−6^) and the differences in beta estimates is significant after Bonferroni correction for the total number of significant genomic loci. (b) Genomic loci associated with anthropometric traits in published genome‐wide association studies (GWAS). Previous associations have been retrieved from the GWAS catalog (https://www.ebi.ac.uk/gwas/). Data extracted from Functional Mapping and Annotation of Genome‐Wide Association Studies (FUMA, http://fuma.ctglab.nl/)
**Table S7**. Heritability as estimated by BOLT‐LMM, v2.3.2, on genotyped single nucleotide polymorphisms (SNPs) on anthropometric traits in the UK Biobank. Variance explained by BOLT‐LMM's linear predictor—using the default mixture‐of‐Gaussians prior on SNP effect sizes, which accounts for larger‐effect SNPs—and variance theoretically explained by an optimal linear predictor, that is, SNP heritability (*h* ^2^g). Additionally, linkage disequilibrium score regression (LDSC) estimates are presented and the difference between BOLT‐LMM and LDSC estimates
**Table S8**. Genetic correlations for sex‐combined anorexia nervosa genome‐wide association study and genetic correlations across body composition, physical activity, and psychiatric and behavioral traits. (a) Genetic correlations for female‐only anorexia nervosa genome‐wide association study and genetic correlations across body composition, physical activity, and psychiatric and behavioral traits

**Appendix S1** Supporting Information

**Appendix S2** Supporting Information

**Appendix S3** Supporting Information
