# Supplementary material for: Genomics of body fat percentage may contribute to sex bias in anorexia nervosa
Source: Am J Med Genet B Neuropsychiatr Genet. 2018 Dec 28;180(6):428–38. doi: 10.1002/ajmg.b.32709 (PMC6751355; doi:10.1002/ajmg.b.32709)
Supplement: Supplementary file 1 — Figure S1 Twin‐based heritabilities (twin‐h 2) of body mass index (BMI) across the lifespan as calculated by ACE models from Silventoinen et al. Blue values represent males and red females with error bars depicting 95% confidence intervals. After the age of 19, heritabilities are represented for a whole decade (Silventoinen et al., 2016, 2017) Figure S2. Analysis workflow chart. AN = anorexia nervosa; BMI = body mass index; GWAS = genome‐wide association study; LDSC = linkage score disequilibrium regression; MAGIC = Meta‐Analyses of Glucose and Insulin‐related traits Consortium; MDD = major depressive disorder; OCD = obsessive–compulsive disorder; PGC = Psychiatric Genomics Consortium; SSGAC = Social Science Genetic Association Consortium; UKB = UK Biobank Figure S3 (a) QQ plot for the body fat percentage (BF%) genome‐wide‐association study (GWAS). (b) QQ plot for the fat‐free mass (FFM) GWAS Figure S4 (a) Manhattan plot of the meta‐analyzed genome‐wide association study (GWAS) of body fat percentage (BF%). The red line represents the genome‐wide significance threshold of 5 × 10^−8^. Chr = chromosome. (b) Manhattan plot of the meta‐analyzed genome‐wide association study (GWAS) of fat‐free mass (FFM). The red line represents the genome‐wide significance threshold of 5 × 10^−8^. Chr = chromosome Figure S5 (a) Partitioned heritability by 10 cell type groups for body fat percentage in females. The black dashed lines at −log10(P) = 2.3 is the cutoff for Bonferroni significance. CNS = central nervous system SNP = single nucleotide polymorphism. (b) Partitioned heritability by 10 cell type groups for body fat percentage in males. The black dashed lines at −log10(P) = 2.3 is the cutoff for Bonferroni significance. CNS = central nervous system, SNP = single nucleotide polymorphism. (c) Partitioned heritability by 10 cell type groups for body fat percentage in the meta‐analyzed GWAS. The black dashed lines at −log10(P) = 2.3 is the cutoff for Bonferroni significance. CNS = centra [file AJMG-180-428-s001.docx]

**Genomics of body fat percentage may contribute to sex bias in anorexia nervosa**

**Hübel et al.**

**Supplementary Figures**

**
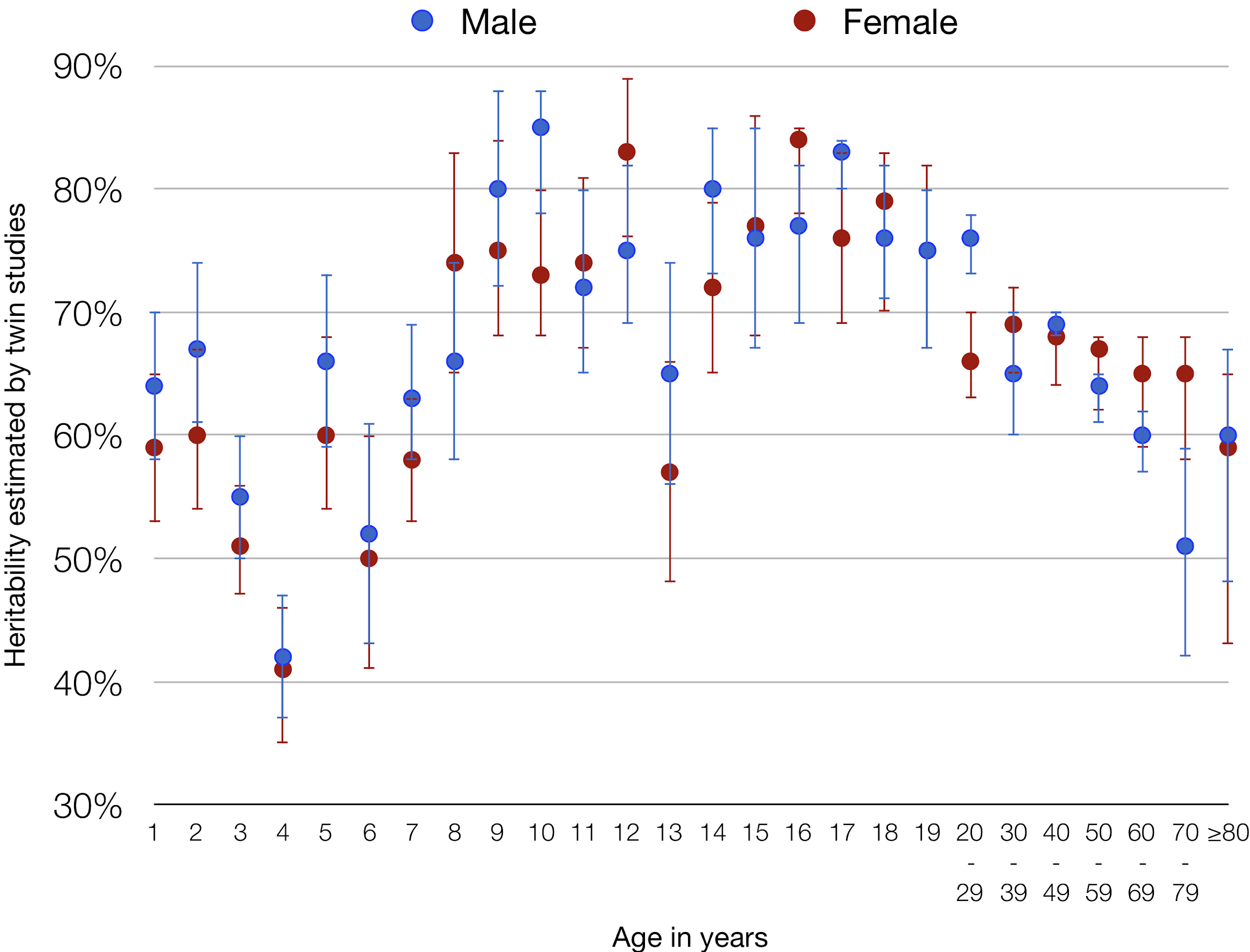
**

**Supplementary Figure 1.** Twin-based heritabilities (*twin-h^2^*) of body mass index (BMI) across the lifespan as calculated by ACE models from Silventoinen et al. Blue values represent males and red females with error bars depicting 95% confidence intervals (CI 95%). After the age of 19 heritabilities are represented for a whole decade [(Silventoinen et al., 2016, 2017)](https://paperpile.com/c/p1LSCa/6kQQ3+meuE).


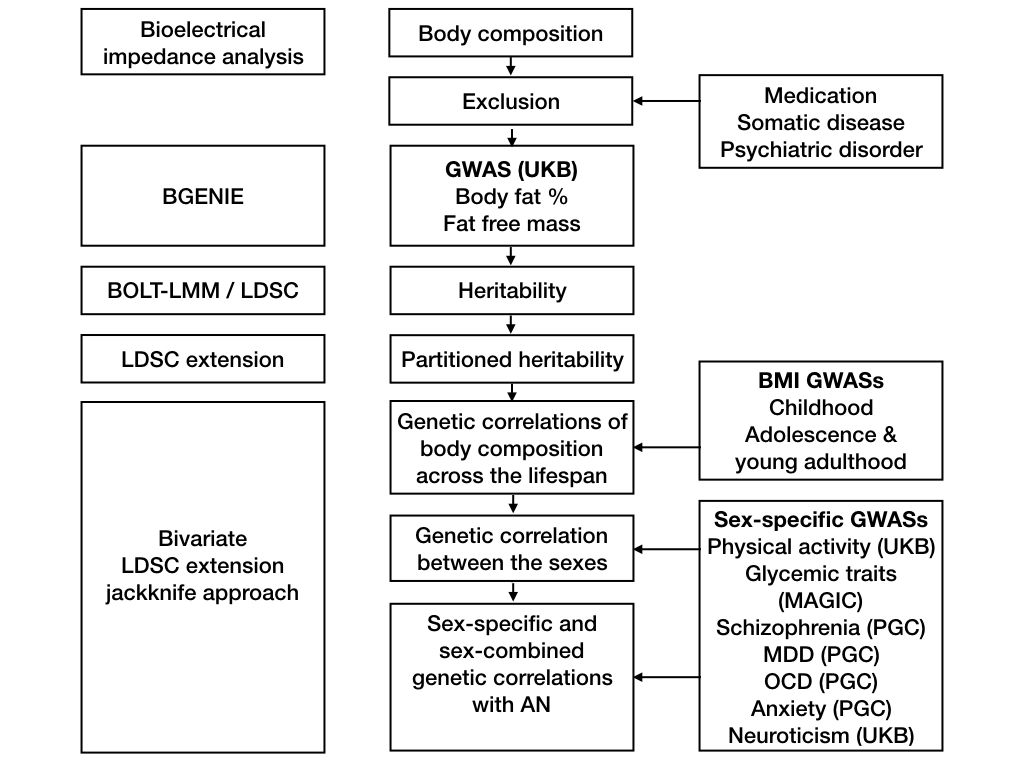


**Supplementary Figure 2.** Analysis workflow chart. AN = anorexia nervosa, BMI = body mass index, GWAS = genome-wide association study, LDSC = linkage score disequilibrium regression, MAGIC = Meta-Analyses of Glucose and Insulin-related traits Consortium, MDD = major depressive disorder, OCD = obsessive-compulsive disorder, PGC = Psychiatric Genomics Consortium, SSGAC = Social Science Genetic Association Consortium, UKB = UK Biobank


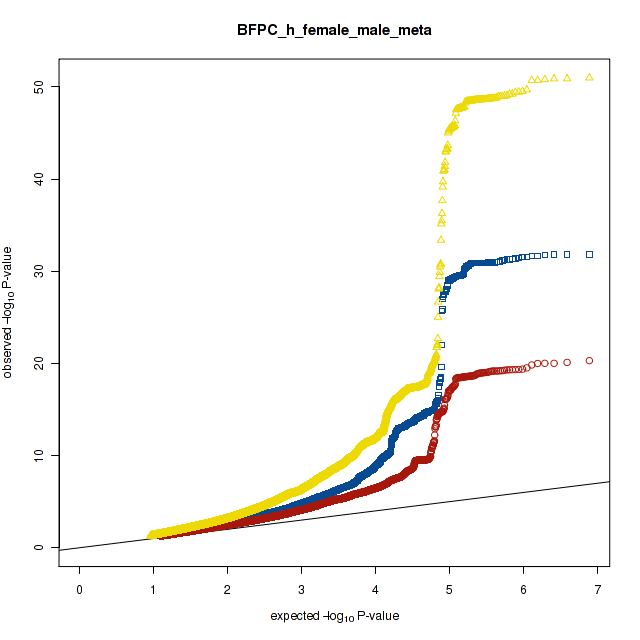


**Supplementary Figure 3a.** QQ plot for the body fat percentage (BF%) genome-wide-association study (GWAS)

The negative logarithm of the observed (y axis) and the expected (x axis) p value is plotted for each single nucleotide polymorphism (SNP; circle, rectangle or triangle), and the black line indicates the null hypothesis of no true association. Different colours depict the three different GWAS: both sexes (yellow), female (red), male (blue). Deviation from the expected p value distribution is evident only in the tail area, suggesting that population stratification was adequately controlled. BPFC = body fat percentage, h = healthy.


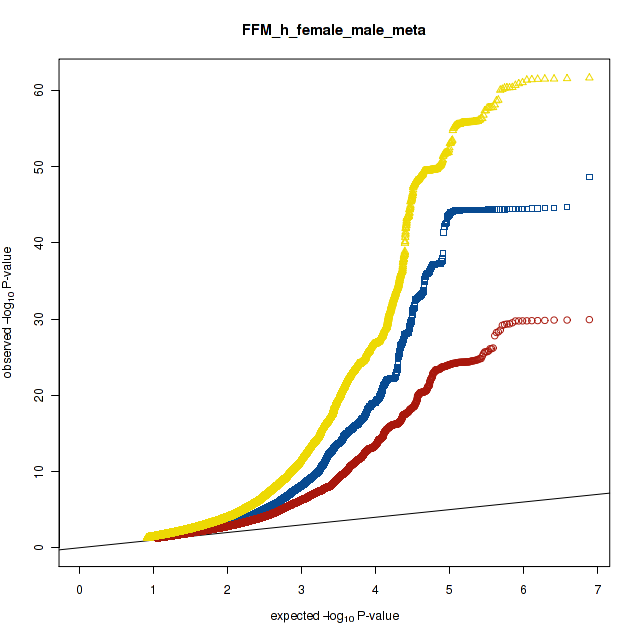


**Supplementary Figure 3b.** QQ plot for the fat free mass (FFM) genome-wide-association study (GWAS)

The negative logarithm of the observed (y axis) and the expected (x axis) p value is plotted for each single nucleotide polymorphism (SNP; circle, rectangle or triangle), and the black line indicates the null hypothesis of no true association. Different colours depict the three different GWAS: both sexes (yellow), female (red), male (blue). Deviation from the expected p value distribution is evident only in the tail area, suggesting that population stratification was adequately controlled. FFM = fat free mass, h = healthy.


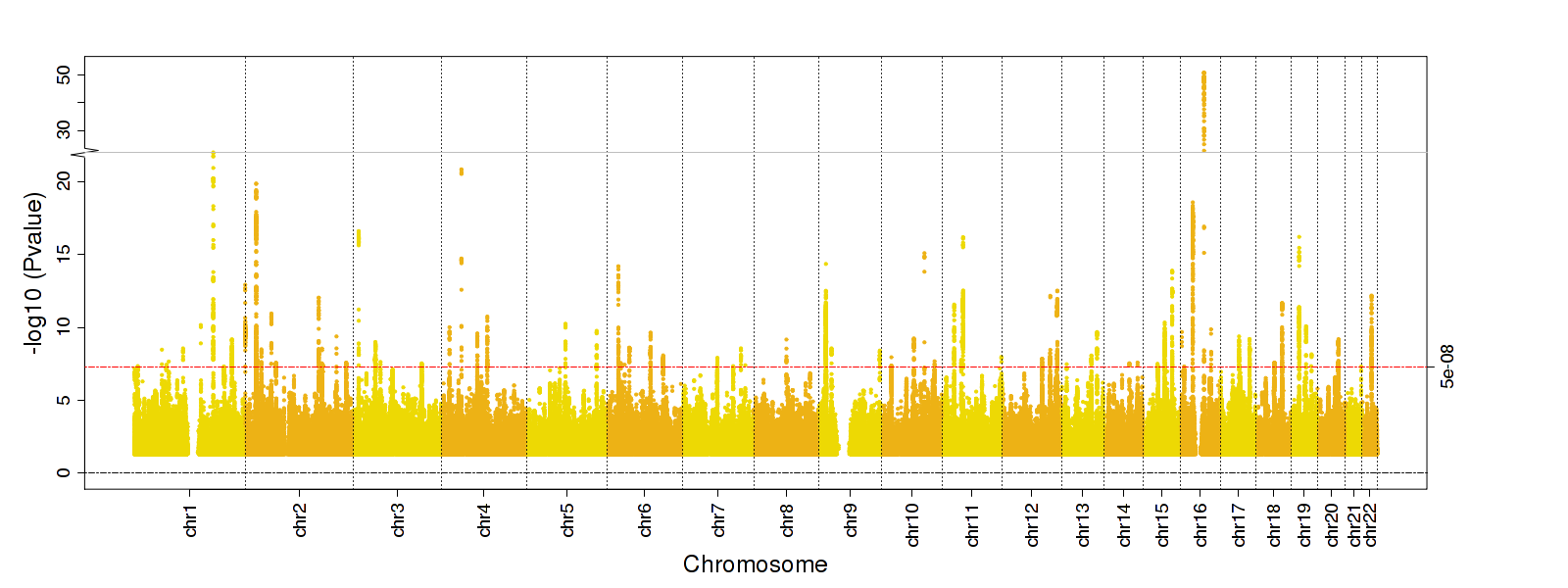


**Supplementary Figure 4a.** Manhattan plot of the meta-analyzed genome-wide association study (GWAS) of body fat percentage (BF%). The red line represents the genome-wide significance threshold of 5x10^-8^. Chr = chromosome.


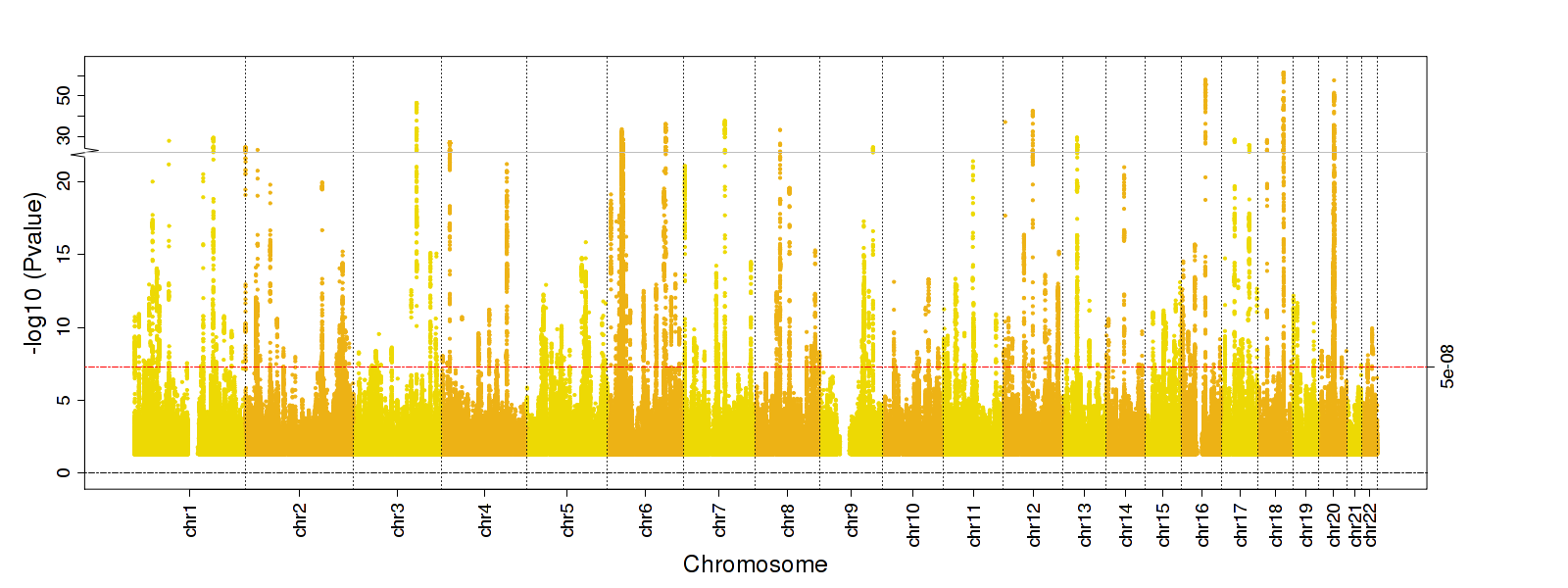


**Supplementary Figure 4b.** Manhattan plot of the meta-analyzed genome-wide association study (GWAS) of fat free mass (FFM). The red line represents the genome-wide significance threshold of 5x10^-8^. Chr = chromosome.


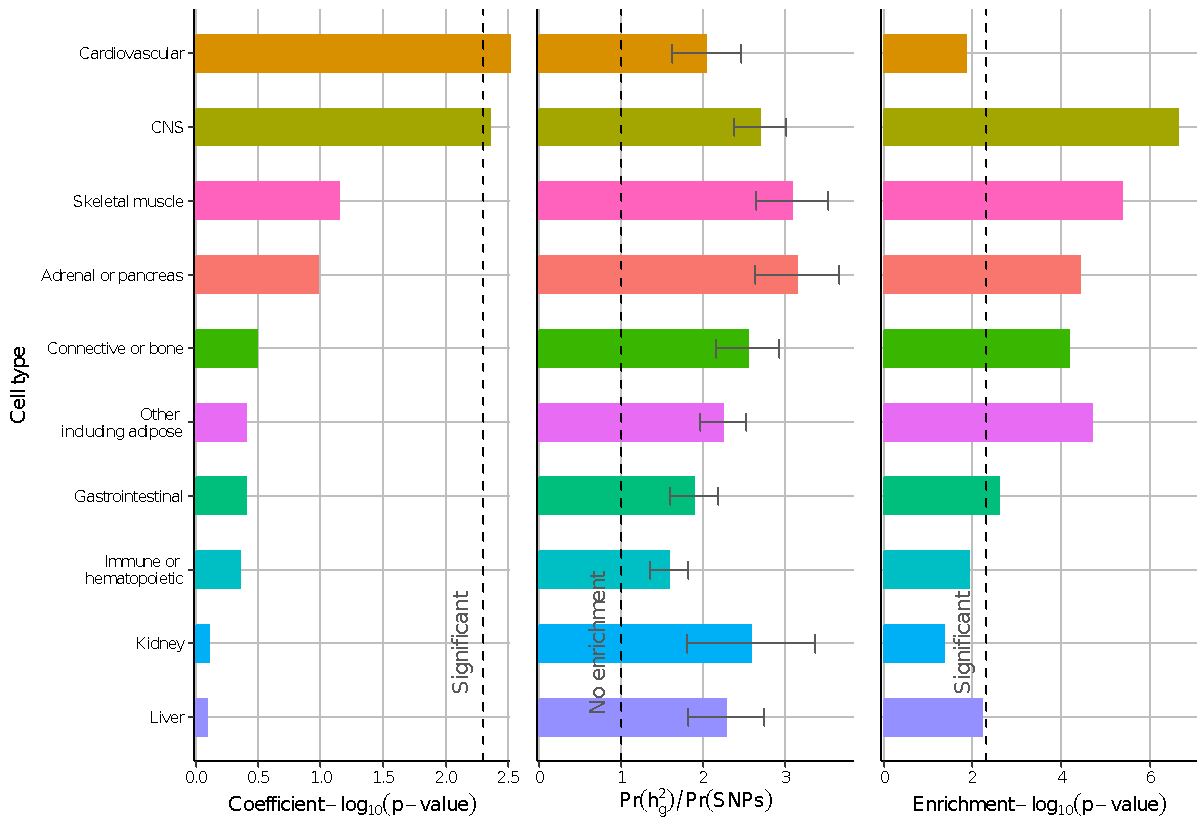


**Supplementary Figure 5a.** Partitioned heritability by 10 cell type groups for body fat percentage in females. The black dashed lines at −log_10_(*P*) = 2.3 is the cutoff for Bonferroni significance. CNS = central nervous system SNP = single nucleotide polymorphism.


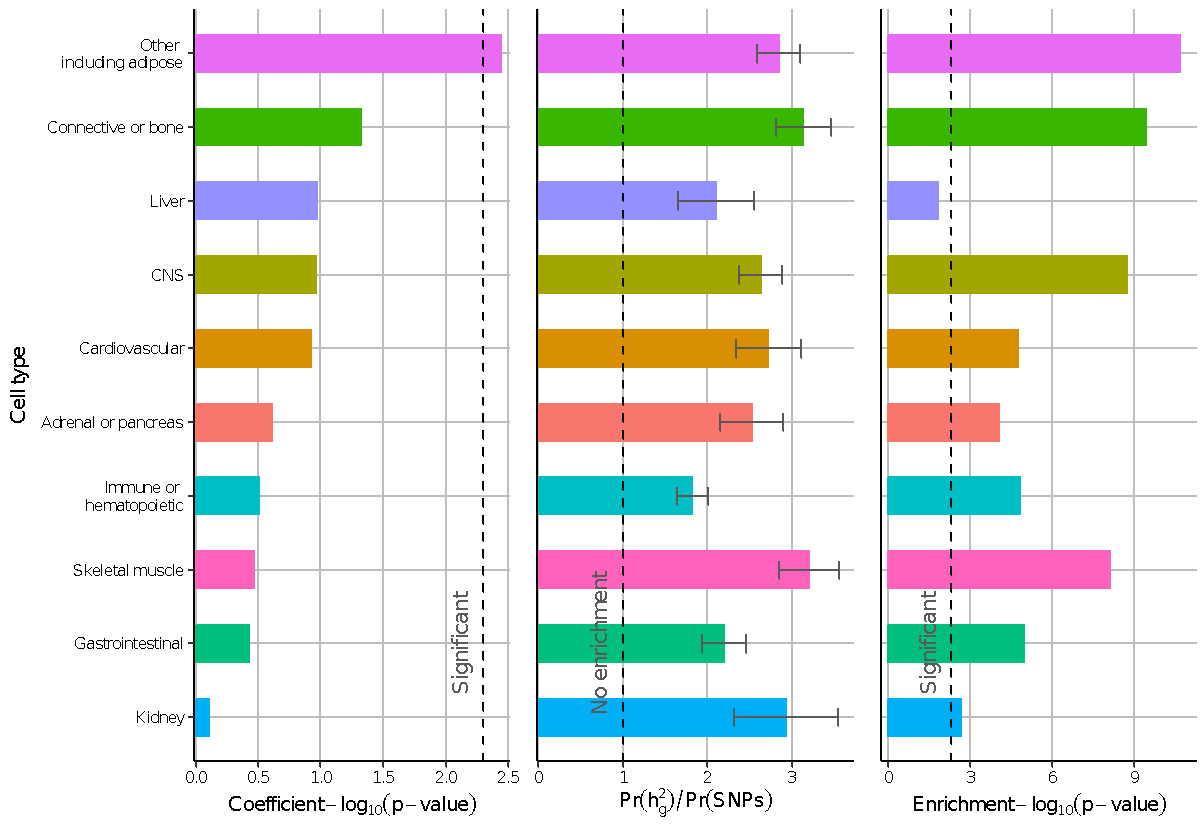


**Supplementary Figure 5b.** Partitioned heritability by 10 cell type groups for body fat percentage in males. The black dashed lines at −log_10_(*P*) = 2.3 is the cutoff for Bonferroni significance. CNS = central nervous system, SNP = single nucleotide polymorphism.

**
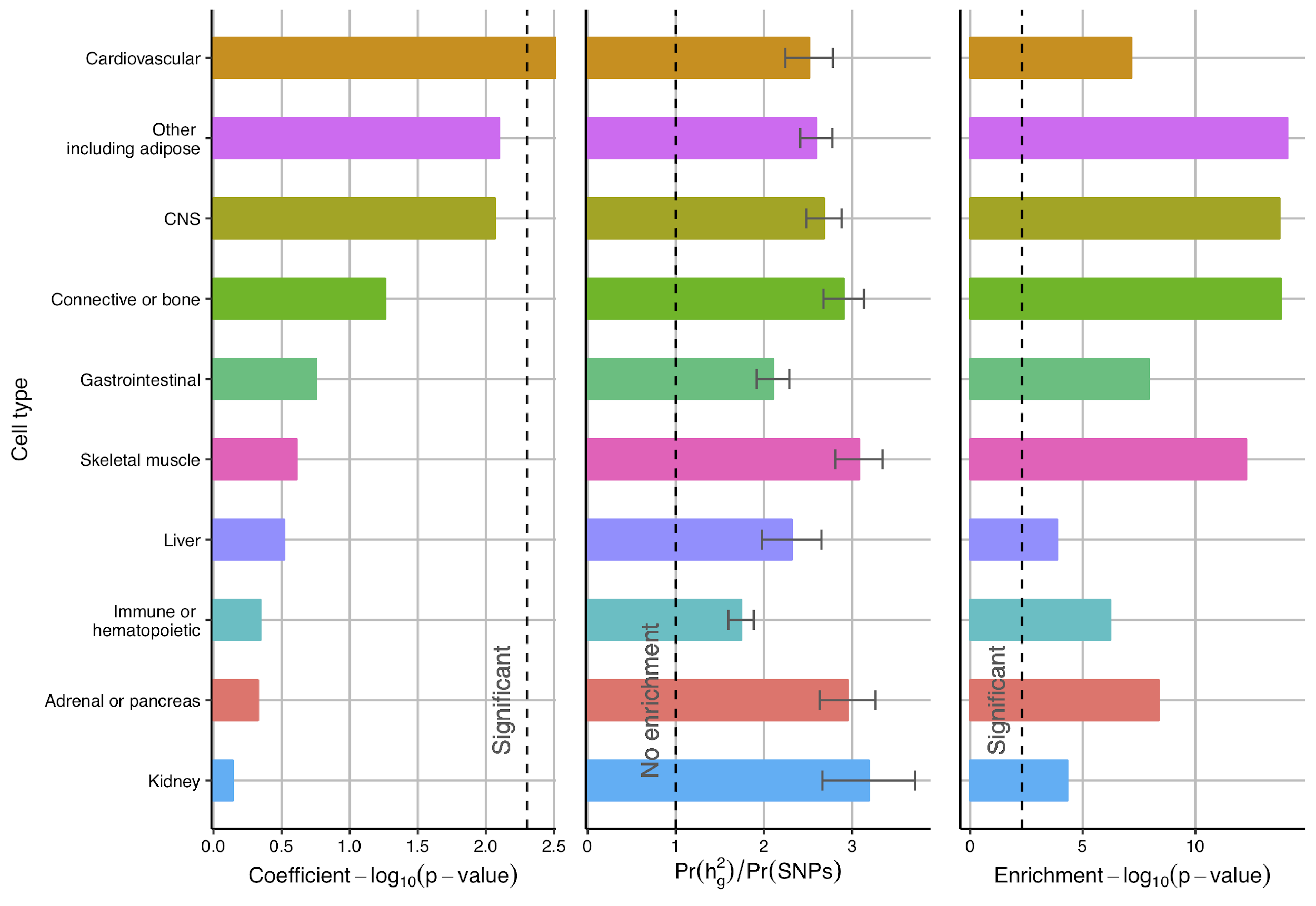
**

**Supplementary Figure 5c.** Partitioned heritability by 10 cell type groups for body fat percentage in the meta-analyzed GWAS. The black dashed lines at −log_10_(*P*) = 2.3 is the cutoff for Bonferroni significance. CNS = central nervous system, SNP = single nucleotide polymorphism.


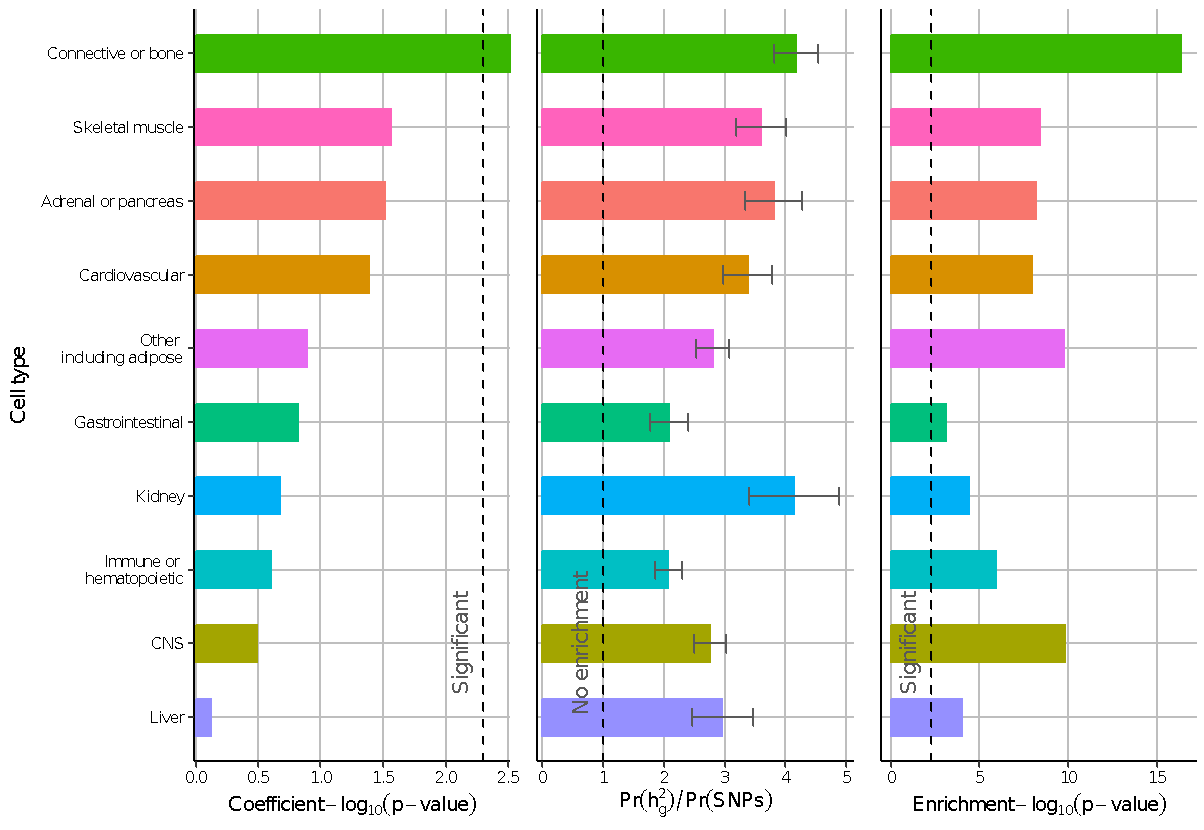


**Supplementary Figure 6a.** Partitioned heritability by 10 cell type groups for fat free mass (FFM) in females. The black dashed lines at −log_10_(*P*) = 2.3 is the cutoff for Bonferroni significance. CNS = central nervous system, SNP = single nucleotide polymorphism.


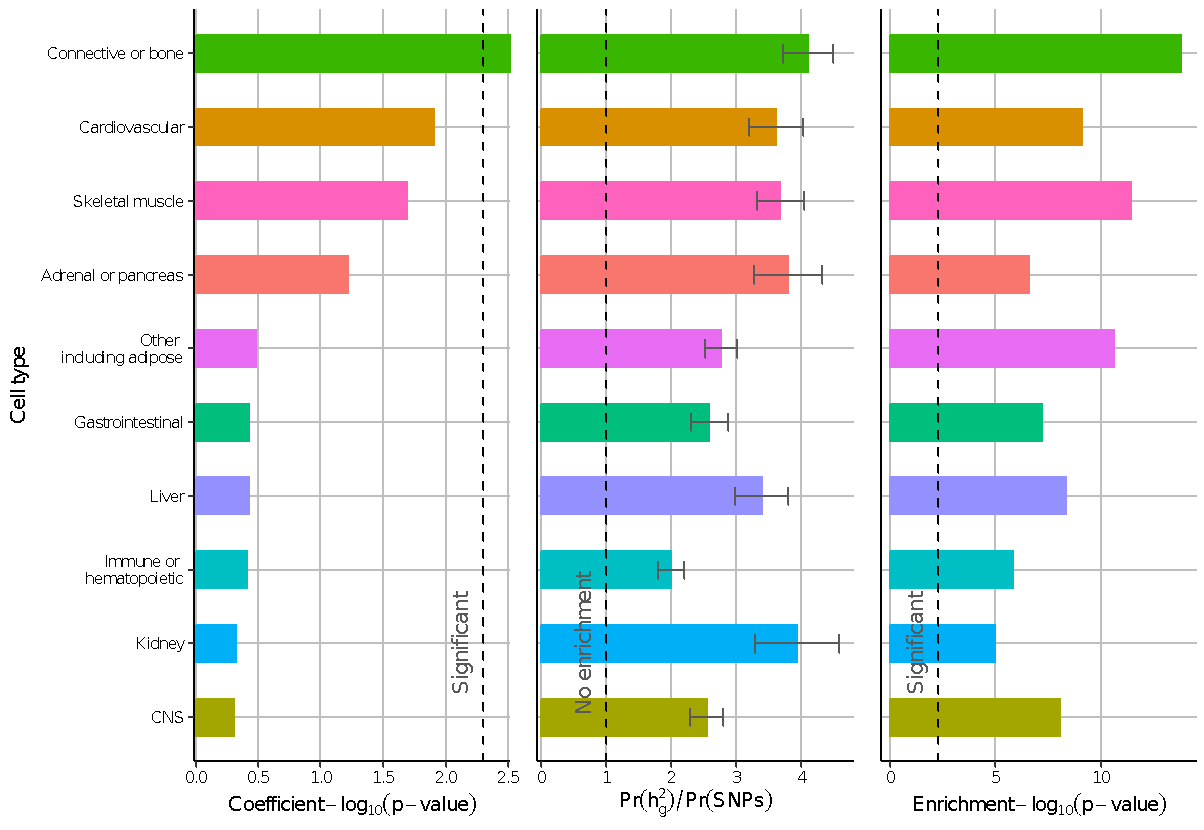


**Supplementary Figure 6b.** Partitioned heritability by 10 cell type groups for fat free mass (FFM) in males. The black dashed lines at −log_10_(*P*) = 2.3 is the cutoff for Bonferroni significance. CNS = central nervous system, SNP = single nucleotide polymorphism.

**
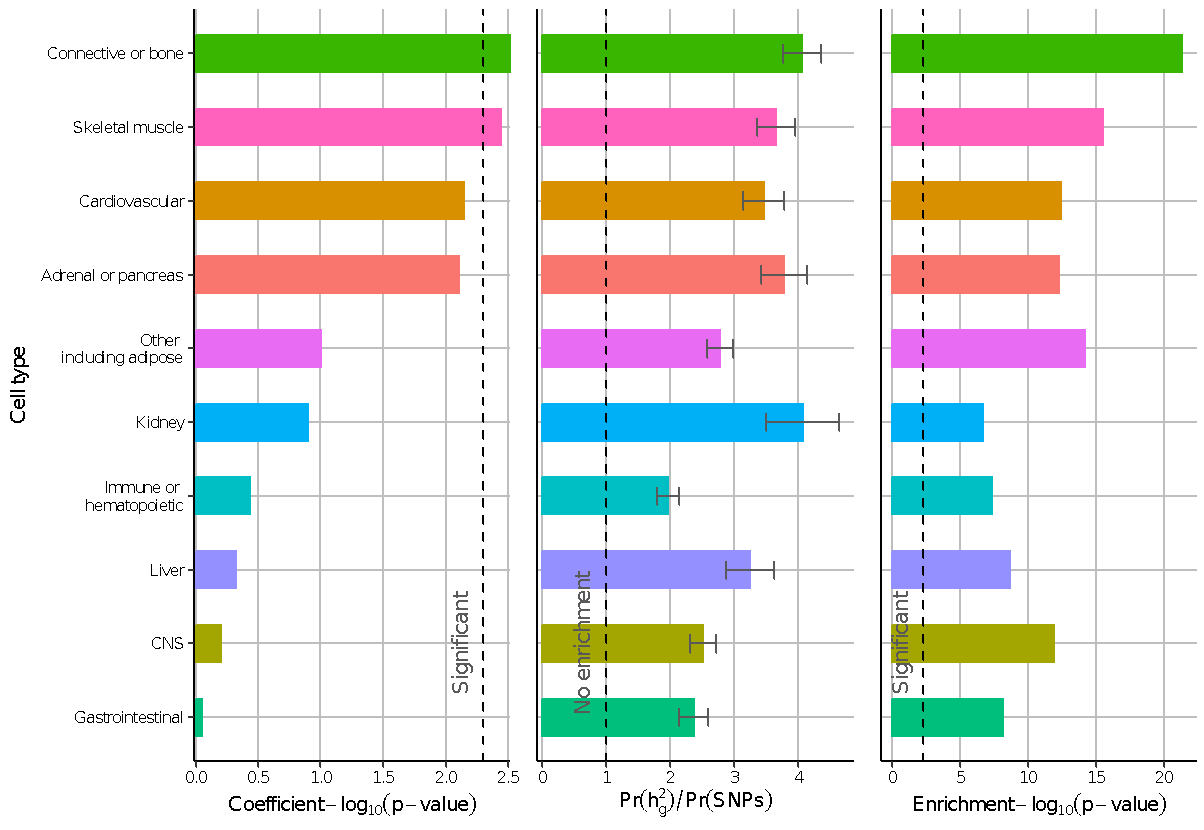
**

**Supplementary Figure 6c.** Partitioned heritability by 10 cell type groups for fat free mass (FFM) in the meta-analyzed GWAS. The black dashed lines at −log_10_(*P*) = 2.3 is the cutoff for Bonferroni significance. CNS = central nervous system, SNP = single nucleotide polymorphism.
