# Supplementary material for: Genomics of body fat percentage may contribute to sex bias in anorexia nervosa
Source: Am J Med Genet B Neuropsychiatr Genet. 2018 Dec 28;180(6):428–38. doi: 10.1002/ajmg.b.32709 (PMC6751355; doi:10.1002/ajmg.b.32709)
Supplement: Supplementary file 3 — Appendix S1 Supporting Information [file AJMG-180-428-s003.docx]

**Genomics of body fat percentage may contribute to sex bias in anorexia nervosa**

**Hübel et al.**

# Supplementary Methods

## **UK Biobank**

UK Biobank ([www.ukbiobank.ac.uk](http://www.ukbiobank.ac.uk)) is a unique epidemiological and prospective resource to generate research findings aimed to improve prevention, diagnosis, and treatment of psychiatric and somatic illnesses. UK Biobank recruited participants from the general population between 2006–2010. All participants were between 40 to 69 years old, were registered with a general practitioner through the United Kingdom’s National Health Service, and lived within traveling distance of one of the assessment centres. UK Biobank is approved by The North West Multi-centre Research Ethics Committee. Genomewide array data for this study were available for 488,363 individuals. Due to this trait-specific medication and illness filtering, the final analysis included 155,961 (45% female) healthy and drug-free European participants which are 32% of the genotyped UK Biobank participants (n = 502,682). This study has been completed under UK Biobank approved study application 16577 and 27546.

## **Body composition**

After the removal of shoes and heavy clothing, body weight was assessed using a Tanita BC-418 MA scale (Tanita Corporation, Arlington Height, IL) (<http://biobank.ctsu.ox.ac.uk/crystal/docs/Bodycomposition.pdf>). Standing height without shoes was assessed with a Seca 202 stadiometer (Seca, Hamburg, Germany). Body mass index (BMI) was calculated by dividing weight in kilograms (kg) by height in metres squared (m^2^). Bioelectrical impedance was measured using the Tanita BC-418 MA scale. Participants stood on the footpads of the analyzer in bare feet while holding the handles. This body composition analyzer calculates fat free mass (FFM) and fat mass (FM) from raw bioelectrical impedance data, using standard formulas including sex, age, height, and athletic or normal mode. We excluded people whose hydration status might be compromised such as by reported alcohol use disorder, metabolic diseases, or by medication (Supplementary Table S1). Bioelectrical impedance analysis (BIA) technology has been extensively validated [(Genton et al., 2003; Kyle et al., 2004)](https://paperpile.com/c/318pmO/Fx3a+w0Wt). Assessing body fat percentage (BF%) by BIA results in more reliable estimates than body mass index (BMI) for healthy individuals [(Mazzoccoli, 2016; Tanamas et al., 2016)](https://paperpile.com/c/318pmO/EyFw+KuC7), is the most feasible in large epidemiological samples, and does not expose participants to radiation.

## **Descriptive statistics and phenotypic associations**

In our descriptive analyses, we choose to report means and standard deviations (SD) for continuous variables and frequencies and percentages for categorical variables. We used the Pearson product moment correlation coefficient to describe associations. We stratified by sex to calculate male- and female-specific genome-wide association studies (GWAS). Analyses were conducted in R version 3.3.2, if not otherwise specified.

## **Residualisation of the phenotype**

We residualized the outcome variables BF% and FFM by performing a multiple regression in R. We included factors related to assessment centre, genotyping batch, smoking status, alcohol consumption, menopause, and continuous measures of age, and socioeconomic status (SES) measured by the Townsend Deprivation Index as independent variables. The Townsend Deprivation Index indicates the level of social deprivation in an area [(Townsend, 1987)](https://paperpile.com/c/318pmO/FN1GV). A higher score indicates a lower average SES. We accounted for underlying population stratification by including the first six ancestry principal components, calculated from genome-wide data on the European subsample using FlashPCA2 [(Abraham, Qiu, & Inouye, 2017)](https://paperpile.com/c/318pmO/MbT0d), in the regression.

## **Power calculations of the genome-wide association studies**

We conducted power calculations for the female and male GWAS using the Genetic Power Calculator [(Purcell, Cherny, & Sham, 2003)](https://paperpile.com/c/318pmO/85abn). Power of 80% at a genome-wide significance threshold of *p*≤5x10^-8^ and a MAF 0.20 to detect a SNP that accounts for 0.1% of trait variance requires 39,580 individuals. According to these results the female and the male GWAS were sufficiently powered to detect genome-wide significant loci with 70,700 females and 85.261 males. With these parameters, the female GWAS had a power of 99.8% and the male GWAS of 99.9%.

## **Genotyping, imputation and quality control**

Blood samples were genotyped on two arrays, which share nearly all of their content: the UKBileve array (*N* = 49,949) or the UK Biobank Axiom array (*N* = 438,414). Genotyping was conducted by Affymetrix and was distributed across 33 different batches of approximately 4,700 samples. UK Biobank provides extensive information on sample processing on its web site,<http://biobank.ctsu.ox.ac.uk/crystal/refer.cgi?id=155583>, and details of the Axiom array are available at<http://media.affymetrix.com/support/downloads/manuals/axiom_2_assay_auto_workflow_user_guide.pdf>. UK Biobank performed stringent quality control on the genotyping data at the Wellcome Trust Centre for Human Genetics (WTCHG). For further details, see:<http://biobank.ctsu.ox.ac.uk/crystal/refer.cgi?id=155580>. Prior to imputation, all variant sites with a call rate below 90% were filtered out. Imputation was carried out by UK Biobank using the IMPUTE3 program and a merged UK10K-1000 Genomes Phase 3 reference panel (details available at [http://biobank.ctsu.ox.ac.uk/crystal/refer.cgi?id=157020)](http://biobank.ctsu.ox.ac.uk/crystal/refer.cgi?id=157020).

We applied additional quality controls. Specifically, we excluded genotyped participants who were pregnant (*N* = 105), had a International Statistical Classification of Diseases and Related Health Problems 10th Revision (ICD-10) [(World Health Organization, 1992)](https://paperpile.com/c/318pmO/zVemE) psychiatric disorder diagnosis described in chapter V (F00–F99) (*N* = 32,510), or self-reported a psychiatric disorder in the mental health questionnaire (*N* = 40,435), or a somatic disease influencing body composition (*N* = 130,663), or were taking medication (*N* = 108,314) that could influence body composition (Supplementary Table S1). Medications were classified using Monthly Index of Medical Specialities (MIMS), British National Formulary (BNF), DrugBank, and Martindale. These numbers do not sum up to the total number excluded as participants could suffer from multiple diseases or were taking multiple medications. Furthermore, we excluded non-European participants identified by k-means clustering (*k* = 4) on the first two principal components derived from the genotype data, and we excluded related individuals (KING relatedness metric >0.088, equivalent to a relatedness value of 0.25; *N* = 7,765) Only participants with complete data were analyzed (*N* = 155,961). SNPs were excluded if they had a minor allele frequency (MAF) smaller than 1%, if no call was made in more than 2% of samples following imputation, if they were imputed with low confidence (INFO<0.8), if they deviated substantially from Hardy-Weinberg equilibrium (HWE test, *p*<10^-7^), or if they were not genotyped and not part of the HRC reference panel [(McCarthy et al., 2016)](https://paperpile.com/c/318pmO/Ih4e7). This left a total of 7,794,483 SNPs for analysis.

## **Genome-wide association studies on body composition measures in UK Biobank**

We calculated sex-specific GWAS on the residualised BF% and FFM, using the program BGENIE v1.2 with the imputed genotype data supplied by UK Biobank (software available at https://jmarchini.org/bgenie/) [(Bycroft et al., 2017)](https://paperpile.com/c/318pmO/ukda). An additive model was used. Furthermore we used the current version of METAL (<http://csg.sph.umich.edu/abecasis/metal/>) to meta-analyze applying a variance-weighted fixed-effect statistical method those sex-specific GWAS [(Willer, Li, & Abecasis, 2010)](https://paperpile.com/c/318pmO/tfX44). Independent loci were estimated by linkage disequilibrium (LD) clumping in Functional Mapping and Annotation [(FUMA; Watanabe, Taskesen, van Bochoven, & Posthuma, 2017)](https://paperpile.com/c/318pmO/a61N/?prefix=FUMA%3B). This reflects the degree to which genome-wide significant hits tag independent regions and/or haplotypes as defined by the LD structure of the data. The most strongly associated SNPs (with a *p* value of < 5x10^−8^) were considered as potential index SNPs. SNPs in LD (*r^2^* > 0.2) with a more strongly associated SNP within 3000kb were assigned to the same locus using Functional Mapping and Annotation [(FUMA; Watanabe et al., 2017)](https://paperpile.com/c/318pmO/a61N/?prefix=FUMA%3B). Overlapping clumps additionally were merged with a second clumping procedure in FUMA merging all lead SNPs with r^2^ = 1 to genomic risk loci. After clumping, independent genome-wide significant loci (5x10^-8^) were compared with entries in the NHGRI-EBI GWAS catalog using FUMA [(MacArthur et al., 2017)](https://paperpile.com/c/318pmO/X6j5z).

## **Genome-wide association study on neuroticism in UK Biobank**

We calculated sex-specific GWASs on neuroticism using the genotype data (prior to imputation) supplied by UK Biobank in males (N = 142,875) and in females (N=144,660; total N = 287,535), following QC as described above. The neuroticism phenotype was calculated as the sum score of neuroticism questions at the baseline assessment [(Smith et al., 2013)](https://paperpile.com/c/318pmO/9z8m), corrected for age and sex-specific means and standard deviations from the UK population [(Eysenck, Eysenck, & Barrett, 1985)](https://paperpile.com/c/318pmO/WeRJ). In a second analysis, individuals were excluded if they reported any psychiatric illness, resulting in 83,413 males and 73,946 females (total N = 157,355; Supplementary Table S1). Sex-stratified linear regressions were performed in PLINK using eight genomic principal components and genotyping batch (as a factor) as covariates and later meta-analyzed using METAL [(Willer et al., 2010)](https://paperpile.com/c/318pmO/tfX44).

**Genome-wide association study on physical activity in the UK Biobank**

We calculated sex-specific genome-wide association analyses of physical activity with imputed genotype data in 29,496 male and 36,758 female (N = 66,254) individuals in the UK Biobank, including age (at recruitment), genotyping array, and genetic principal components 1–20 as covariates. Physical activity in the UK Biobank was measured continuously over a period of 7 days with a wrist-worn accelerometer. General physical activity quality control of raw data is described in detail elsewhere [(Doherty et al., 2017)](https://paperpile.com/c/318pmO/2r2N). We used a wear-time adjusted 7-day average measure of activity, including only individuals meeting UKB QC criterion: good wear-time, good calibration, calibration on own data, and no problem indicators. Our analyses were performed on the intersection of this UK Biobank subset with those passing general genotyping QC: in white British ancestry subset, used in the calculation of ancestry principal components, without excess relatives in the UK Biobank sample, no putative sex chromosome aneuploidy, and were not outliers for heterozygosity and genotype missingness. General genotyping considerations, raw data QC, and imputation procedure in the UK Biobank are described in detail elsewhere [(Bycroft et al., 2017)](https://paperpile.com/c/318pmO/ukda).

**Genome-wide association study on anxiety in the UK Biobank**

We calculated genome-wide association analyses of anxiety disorders with imputed genotype data on 25,443 cases compared to 58,113 controls [(Purves et al., 2017)](https://paperpile.com/c/318pmO/jRPF). Cases met criteria for probable lifetime anxiety disorder diagnosis if they either self-reported a professional diagnosis of any of the five core anxiety disorders (generalized anxiety disorder, panic disorder, specific phobia, agoraphobia, or social anxiety disorder) or met criteria for probable lifetime generalized anxiety disorder according to the Composite International Diagnostic Interview question set [(Davis et al., 2018; Kessler, Andrews, Mroczek, Ustun, & Wittchen, 1998)](https://paperpile.com/c/318pmO/ER1O+TTQC). Cases did not report a diagnosis of schizophrenia, psychosis, ADHD, autism, any eating disorder, or bipolar disorder. Controls were screened for any evidence of psychiatric or substance use disorders. Participants were limited to individuals of white European ancestry, who were not excessively related, had no putative sex chromosome aneuploidy, and were not outliers for heterozygosity and genotype missingness. Stratified linear regressions were performed on ~7 million SNPs of high imputation quality (INFO > 0.9) with a minimum minor allele frequency of 1% in BGENIE v1.2 controlling for six genetic principal components, assessment center, genotyping batch, and age.

## **Genetic correlations**

Using an analytic extension of LDSC, we calculated genetic correlations (*SNP-r_g_*) across the computed GWAS and with a wide range of metabolic measures, such as body composition, insulin sensitivity, fasting glucose and insulin, physical activity, and psychiatric traits and disorders, such as depression and neuroticism. For some of these traits, sex-specific summary statistics were available. *SNP-r_g_* capture the degree to which traits or disorders share common genetic variants and here are estimated using the pattern of LD across the genome. The contribution of any SNP to the phenotype comprises both its own contribution, and the contribution of all variants with which it is in LD. SNPs in regions of high LD are thus more likely to be in LD with a variant that has a true effect on the trait of interest. SNPs in higher LD, therefore, represent a greater proportion of variance in the phenotype on average, than SNPs in regions of lower LD. The *SNP-r_g_* were corrected for multiple comparisons after assessing the number of independent tests by matrix decomposition. Furthermore, we tested whether the *SNP-r_g_* between the females and males GWAS were significantly different from 1 calculating a standard error using a block jackknife method [(Bulik-Sullivan, Finucane, et al., 2015; H. Finucane, personal communication, 2017)](https://paperpile.com/c/318pmO/JWeN/?suffix=%3B%20H.%20Finucane%2C%20personal%20communication%2C%202017).

## **Matrix decomposition to identify number of independent tests**

We used a Bonferroni threshold (by correcting α as α/*N*) to estimate the number of significant genetic correlations, with *N* = estimated number of independent tests. We built a similarity matrix reflecting the trait similarity where *D =* number of principal components accounting for 99.5% of the data variance in the genetic correlation matrix. In that case, *D* is the estimated number of independent traits (GWAS), and the number of independent tests can be computed as *N* = (*D*(*D*-1))/2.

**Sex-specific analyses of genetic determinants of body composition**

We investigated differences between females and males in heritability, *SNP-h^2^*, and genetic architecture, *SNP-r_g_*. We calculated differences (d) in the *SNP-h^2^* estimates between males and females and their standard errors. We calculated the *SNP-r_g_* of female and male GWASs, and tested whether this was different from 1 to identify potential genetic heterogeneity between the sexes using the same approach. We calculated the *SNP-r_g_* of the female and male GWAS with AN separately to investigate the relationship of the heterogeneity with the risk for AN. To test the statistical significance of all estimates, we calculated their standard error and corresponding *p* value by applying a block jackknife method, as described and implemented in ldsc v1.0.0 by Bulik-Sullivan et al. [(2015](https://paperpile.com/c/318pmO/C1mgM/?noauthor=1)).

**Block jackknife method**

*Comparing two genetic correlations using jackknife and ldsc*

Let there be four phenotypes A, B, C, and D. The goal is to compare the genetic correlation between A and B to the genetic correlation between C and D. Global estimates of these correlations can be computed using the ldsc software and will be noted r(A,B) and r(C,D). The same software can output jackknife delete values for genetic covariance: G(A,B), G(C,D), as well as for heritability: H(A,B) and H(C,D). These jackknife delete values are estimated by excluding blocks of values (here, number of blocks n = 200). The n-dimensional vectors G(A,B), G(C,D), H(A,B) and H(C,D) can be used to generate genetic correlation delete values R(A,B) and R(C,D). The difference between the global estimates r(A,B) and r(C,D) is d(AB,CD), and the difference between the vectors R(A,B) and R(C,D) is D(AB,CD). The global genetic correlation difference d(AB,CD) and the delete values D(AB,CD) are used to compute jackknife pseudovalues. The ith pseudovalue is:

$$P_{i}(AB,CD)=n\times d(AB,CD)-(n-1)*P_{i}(AB,CD)$$

The mean and variance of the jackknife pseudovalues are:

$$m(AB,CD)=\frac{1}{n}\sum_{i=1}^{n} P_{i}(AB,CD)$$

$$v(AB,CD)=\frac{1}{n-1}\sum_{i=1}^{n} {(P_{i}(AB,CD)-m(AB,CD))}^{2}$$

The jackknife estimate of the difference between the two correlations m(AB,CD) can then be compared to test H_0_ : θ = θ_0_ (θ_0_ = 0 for no difference between genetic correlations), and a p-value can be derived from the z statistic:

$$z(AB,CD)=\frac{m(AB,CD)-\theta_{0}}{\sqrt{(1/n)\times v(AB,CD)}}$$

**References**

[Abraham, G., Qiu, Y., & Inouye, M. (2017). FlashPCA2: principal component analysis of Biobank-scale genotype datasets. *Bioinformatics* , *33*(17), 2776–2778. https://doi.org/](http://paperpile.com/b/318pmO/MbT0d)[10.1093/bioinformatics/btx299](http://dx.doi.org/10.1093/bioinformatics/btx299)

[Bulik-Sullivan, B. K., Finucane, H. K., Anttila, V., Gusev, A., Day, F. R., Loh, P.-R., … Neale. (2015). An atlas of genetic correlations across human diseases and traits. *Nature Genetics*, *47*(11), 1236–1241. https://doi.org/](http://paperpile.com/b/318pmO/JWeN)[10.1038/ng.3406](http://dx.doi.org/10.1038/ng.3406)

[Bulik-Sullivan, B. K., Loh, P. R., Finucane, H. K., Ripke, S., Yang, J., Schizophrenia Working Group of the Psychiatric Genomics Consortium, … Neale, B. M. (2015). LD Score regression distinguishes confounding from polygenicity in genome-wide association studies. *Nature Genetics*, *47*(3), 291–295. https://doi.org/](http://paperpile.com/b/318pmO/C1mgM)[10.1038/ng.3211](http://dx.doi.org/10.1038/ng.3211)

[Bycroft, C., Freeman, C., Petkova, D., Band, G., Elliott, L. T., Sharp, K., … Marchini, J. (2017). *Genome-wide genetic data on ~500,000 UK Biobank participants*. *bioRxiv*. https://doi.org/](http://paperpile.com/b/318pmO/ukda)[10.1101/166298](http://dx.doi.org/10.1101/166298)

[Davis, K. A. S., Coleman, J. R. I., Adams, M., Allen, N., Breen, G., Cullen, B., … Hotopf, M. (2018). Mental health in UK Biobank: development, implementation and results from an online questionnaire completed by 157 366 participants. *BJPsych Open*, *4*(3), 83–90. https://doi.org/](http://paperpile.com/b/318pmO/TTQC)[10.1192/bjo.2018.12](http://dx.doi.org/10.1192/bjo.2018.12)

[Doherty, A., Jackson, D., Hammerla, N., Plötz, T., Olivier, P., Granat, M. H., … Wareham, N. J. (2017). Large Scale Population Assessment of Physical Activity Using Wrist Worn Accelerometers: The UK Biobank Study. *PloS One*, *12*(2), e0169649. https://doi.org/](http://paperpile.com/b/318pmO/2r2N)[10.1371/journal.pone.0169649](http://dx.doi.org/10.1371/journal.pone.0169649)

[Eysenck, S. B. G., Eysenck, H. J., & Barrett, P. (1985). A revised version of the psychoticism scale. *Personality and Individual Differences*, *6*(1), 21–29. https://doi.org/](http://paperpile.com/b/318pmO/WeRJ)[10.1016/0191-8869(85)90026-1](http://dx.doi.org/10.1016/0191-8869(85)90026-1)

[Genton, L., Hans, D., Karsegard, V. L., Kyle, U. G., Slosman, D. O., & Pichard, C. (2003). Comparison of dual energy X-ray absorptiometry (DXA) with bioelectrical impedance analysis (BIA) in obese women. *Clinical Nutrition* , *22*, S1. https://doi.org/](http://paperpile.com/b/318pmO/w0Wt)[10.1016/s0261-5614(03)80002-x](http://dx.doi.org/10.1016/s0261-5614(03)80002-x)

[Kessler, R. C., Andrews, G., Mroczek, D., Ustun, B., & Wittchen, H.-U. (1998). The World Health Organization Composite International Diagnostic Interview short-form (CIDI-SF). *International Journal of Methods in Psychiatric Research*, *7*(4), 171–185. https://doi.org/](http://paperpile.com/b/318pmO/ER1O)[10.1002/mpr.47](http://dx.doi.org/10.1002/mpr.47)

[Kyle, U. G., Bosaeus, I., De Lorenzo, A. D., Deurenberg, P., Elia, M., Manuel Gómez, J., … ESPEN. (2004). Bioelectrical impedance analysis-part II: utilization in clinical practice. *Clinical Nutrition* , *23*(6), 1430–1453. https://doi.org/](http://paperpile.com/b/318pmO/Fx3a)[10.1016/j.clnu.2004.09.012](http://dx.doi.org/10.1016/j.clnu.2004.09.012)

[MacArthur, J., Bowler, E., Cerezo, M., Gil, L., Hall, P., Hastings, E., … Parkinson, H. (2017). The new NHGRI-EBI Catalog of published genome-wide association studies (GWAS Catalog). *Nucleic Acids Research*, *45*(D1), D896–D901. https://doi.org/](http://paperpile.com/b/318pmO/X6j5z)[10.1093/nar/gkw1133](http://dx.doi.org/10.1093/nar/gkw1133)

[Mazzoccoli, G. (2016). Body composition: Where and when. *European Journal of Radiology*, *85*(8), 1456–1460. https://doi.org/](http://paperpile.com/b/318pmO/KuC7)[10.1016/j.ejrad.2015.10.020](http://dx.doi.org/10.1016/j.ejrad.2015.10.020)

[McCarthy, S., Das, S., Kretzschmar, W., Delaneau, O., Wood, A. R., Teumer, A., … Haplotype Reference Consortium. (2016). A reference panel of 64,976 haplotypes for genotype imputation. *Nature Genetics*, *48*(10), 1279–1283. https://doi.org/](http://paperpile.com/b/318pmO/Ih4e7)[10.1038/ng.3643](http://dx.doi.org/10.1038/ng.3643)

[Purcell, S., Cherny, S. S., & Sham, P. C. (2003). Genetic Power Calculator: design of linkage and association genetic mapping studies of complex traits. *Bioinformatics* , *19*(1), 149–150. Retrieved from](http://paperpile.com/b/318pmO/85abn) <https://www.ncbi.nlm.nih.gov/pubmed/12499305>

[Purves, K. L., Coleman, J. R. I., Rayner, C., Hettema, J. M., Deckert, J., McIntosh, A. M., … Eley, T. C. (2017). *The Common Genetic Architecture of Anxiety Disorders*. *bioRxiv*. https://doi.org/](http://paperpile.com/b/318pmO/jRPF)[10.1101/203844](http://dx.doi.org/10.1101/203844)

[Smith, D. J., Nicholl, B. I., Cullen, B., Martin, D., Ul-Haq, Z., Evans, J., … Pell, J. P. (2013). Prevalence and characteristics of probable major depression and bipolar disorder within UK biobank: cross-sectional study of 172,751 participants. *PloS One*, *8*(11), e75362. https://doi.org/](http://paperpile.com/b/318pmO/9z8m)[10.1371/journal.pone.0075362](http://dx.doi.org/10.1371/journal.pone.0075362)

[Tanamas, S. K., Lean, M. E. J., Combet, E., Vlassopoulos, A., Zimmet, P. Z., & Peeters, A. (2016). Changing guards: time to move beyond body mass index for population monitoring of excess adiposity. *QJM: Monthly Journal of the Association of Physicians*, *109*(7), 443–446. https://doi.org/](http://paperpile.com/b/318pmO/EyFw)[10.1093/qjmed/hcv201](http://dx.doi.org/10.1093/qjmed/hcv201)

[Townsend, P. (1987). Deprivation. *Journal of Social Policy*, *16*(02), 125. https://doi.org/](http://paperpile.com/b/318pmO/FN1GV)[10.1017/s0047279400020341](http://dx.doi.org/10.1017/s0047279400020341)

[Watanabe, K., Taskesen, E., van Bochoven, A., & Posthuma, D. (2017). Functional mapping and annotation of genetic associations with FUMA. *Nature Communications*, *8*(1), 1826. https://doi.org/](http://paperpile.com/b/318pmO/a61N)[10.1038/s41467-017-01261-5](http://dx.doi.org/10.1038/s41467-017-01261-5)

[Willer, C. J., Li, Y., & Abecasis, G. R. (2010). METAL: fast and efficient meta-analysis of genomewide association scans. *Bioinformatics* , *26*(17), 2190–2191. https://doi.org/](http://paperpile.com/b/318pmO/tfX44)[10.1093/bioinformatics/btq340](http://dx.doi.org/10.1093/bioinformatics/btq340)

[World Health Organization. (1992). *ICD-10: International Statistical Classification of Diseases and Related Health Problems: 10th revision*. Geneva: World Health Organization.](http://paperpile.com/b/318pmO/zVemE)
