# Supplementary material for: Genomics of body fat percentage may contribute to sex bias in anorexia nervosa
Source: Am J Med Genet B Neuropsychiatr Genet. 2018 Dec 28;180(6):428–38. doi: 10.1002/ajmg.b.32709 (PMC6751355; doi:10.1002/ajmg.b.32709)
Supplement: Supplementary file 4 — Appendix S2 Supporting Information [file AJMG-180-428-s004.docx]

Hunna J Watson PhD, MPsychClin, MBiostats^1,2,3^, Zeynep Yilmaz PhD^1,4,a^, Laura M Thornton PhD^1,a^, Christopher Hübel MD, MSc^5,6,a^, Jonathan RI Coleman PhD^5,7,a^, Héléna A Gaspar PhD^5,7,b^, Julien Bryois PhD^6,b^, Anke Hinney PhD^8,b^, Virpi M Leppä PhD^6^, Manuel Mattheisen MD^9,10,11,12,b^, Sarah E Medland PhD^13,b^, Stephan Ripke MD, PhD^14,15,16,b^, Shuyang Yao PhD^6,b^, Paola Giusti-Rodríquez PhD^4^, Anorexia Nervosa Genetics Initiative, Ken B Hanscombe PhD^17^, Kirstin L Purves MSc^5^, Eating Disorders Working Group of the Psychiatric Genomics Consortium (PGC-ED), Roger AH Adan PhD^18,19,20^, Lars Alfredsson PhD^21^, Tetsuya Ando MD, PhD^22^, Ole A Andreassen MD, PhD^23^, Jessica H Baker PhD^1^, Wade H Berrettini MD, PhD^24^, Ilka Boehm PhD^25^, Claudette Boni PhD^26^, Vesna Boraska Perica PhD^27,28^, Katharina Buehren MD, PhD^29^, Roland Burghardt MD^30^, Matteo Cassina MD^31^, Sven Cichon PhD^32^, Maurizio Clementi MD^31^, Roger D Cone PhD^33^, Philippe Courtet MD^34^, Scott Crow MD^35^, James J Crowley PhD^4,10^, Unna N Danner PhD^19^, Oliver SP Davis MSc, PhD^36,37^, Martina de Zwaan MD^38^, George Dedoussis PhD^39^, Daniela Degortes PhD^40^, Janiece E DeSocio PhD, RN, PMHNP-BC^41^, Danielle M Dick PhD^42^, Dimitris Dikeos MD^43^, Christian Dina PhD^44,45^, Monika Dmitrzak-Weglarz PhD^46^, Elisa Docampo Martinez MD, PhD^47,48,49^, Laramie E Duncan PhD^50^, Karin Egberts MD^51^, Stefan Ehrlich MD^25^, Geòrgia Escaramís PhD^47,48,49^, Tõnu Esko PhD^52,53^, Xavier Estivill MD, PhD^47,48,49,54^, Anne Farmer MD^5^, Angela Favaro MD, PhD^40^, Fernando Fernández-Aranda PhD^55,56^, Manfred M Fichter MD, Dipl-Psych^57,58^, Krista Fischer PhD^52^, Manuel Föcker MD^8^, Lenka Foretova MD, PhD^59^, Andreas J Forstner MD^32,60,61,62,63^, Monica Forzan PhD^31^, Christopher S Franklin PhD^27^, Steven Gallinger MD^64^, Ina Giegling PhD^65^, Johanna Giuranna MSc^8^, Fragiskos Gonidakis MD^66^, Philip Gorwood MD, PhD^26,67^, Monica Gratacos Mayora MD, PhD^47,48,49^, Sébastien Guillaume MD, PhD^34^, Yiran Guo PhD^68^, Hakon Hakonarson MD, PhD^68,69^, Konstantinos Hatzikotoulas MD, PhD^27^, Joanna Hauser MD, PhD^70^, Johannes Hebebrand MD^8^, Sietske G Helder PhD^5,71^, Stefan Herms MSc^32,63^, Beate Herpertz-Dahlmann MD^29^, Wolfgang Herzog MD^72^, Laura M Huckins PhD^27,73^, James I Hudson MD, ScD^74^, Hartmut Imgart MD^75^, Hidetoshi Inoko PhD^76^, Vladimir Janout PhD^77^, Susana Jiménez-Murcia PhD^55,56^, Antonio Julià PhD^78^, Gursharan Kalsi PhD^5^, Deborah Kaminská PhD^79^, Jaakko Kaprio MD, PhD^80,81^, Leila Karhunen PhD^82^, Andreas Karwautz MD^83^, Martien JH Kas PhD^18,84^, James L Kennedy MD, FRCP(C)^85^, Anna Keski-Rahkonen MD, PhD, MPH^80^, Kirsty Kiezebrink PhD, FHEA, RNutr^86^, Youl-Ri Kim MD, PhD^87^, Lars Klareskog MD^88^, Kelly L Klump PhD^89^, Gun Peggy S Knudsen PhD^90^, Maria C La Via MD^1^, Stephanie Le Hellard PhD^91,92,93^, Robert D Levitan MD^94^, Dong Li PhD^68^, Lisa Lilenfeld PhD^95^, Bochao Danae Lin PhD^18^, Jolanta Lissowska Ph.D.^96^, Jurjen Luykx MD PhD^18^, Pierre J Magistretti MD, PhD^97,98^, Mario Maj MD, PhD^99^, Katrin Mannik PhD^52,100^, Sara Marsal MD, PhD^78^, Christian R Marshall PhD^101^, Morten Mattingsdal PhD^23^, Sara McDevitt MB, MD, MRCPsych, MMedED^102,103^, Peter McGuffin MD^5^, Andres Metspalu PhD, MD^52,104^, Ingrid Meulenbelt PhD^105^, Nadia Micali MD, PhD^106,107^, Karen Mitchell PhD^108^, Alessio Maria Monteleone MD^99^, Palmiero Monteleone MD^109^, Melissa A Munn-Chernoff PhD^1^, Benedetta Nacmias PhD^110^, Marie Navratilova MUDr., PhD^59^, Ioanna Ntalla PhD^39^, Julie K O’Toole MD^111^, Roel A Ophoff PhD^18,112^, Leonid Padyukov MD, PhD^88^, Aarno Palotie MD, PhD^53,81,113^, Jacques Pantel PhD^26^, Hana Papezova MD, PhD^79^, Dalila Pinto PhD^73^, Raquel Rabionet PhD^114,115,116^, Anu Raevuori MD, PhD^80^, Nicolas Ramoz PhD^26^, Ted Reichborn-Kjennerud MD, PhD^90,117^, Valdo Ricca MD^110,118^, Samuli Ripatti PhD^119^, Franziska Ritschel MSc^25,120^, Marion Roberts PhD^5^, Alessandro Rotondo MD^121^, Dan Rujescu MD^57,65^, Filip Rybakowski MD, PhD^122^, Paolo Santonastaso MD^123^, André Scherag PhD^124^, Stephen W Scherer PhD, FRSC^125^, Ulrike Schmidt MD, PhD^5^, Nicholas J Schork PhD^126^, Alexandra Schosser PhD^127^, Jochen Seitz MD^29^, Lenka Slachtova PhD^128^, P. Eline Slagboom PhD^105^, Margarita CT Slof-Op ‘t Landt PhD^129,130^, Agnieszka Slopien MD, PhD^131^, Sandro Sorbi MD^110,132^, Beata ŚwiątkowskaPhD^133^, Jin P Szatkiewicz PhD^4^, Ioanna Tachmazidou PhD^27^, Elena Tenconi MD^40^, Alfonso Tortorella MD^134,135^, Federica Tozzi MD^136^, Janet Treasure PhD, FRCP, FRCPsych^5^, Artemis Tsitsika MD, PhD^137^, Marta Tyszkiewicz-Nwafor MD, PhD^122^, Konstantinos Tziouvas MD, MSc^138^, Annemarie A van Elburg MD, PhD^19,139^, Eric F van Furth PhD^129,130^, Gudrun Wagner Dr, MSc, DPO^83^, Esther Walton Dr. rer. nat., PhD^25^, Elisabeth Widen MD, PhD^81^, Eleftheria Zeggini PhD^27,140^, Stephanie Zerwas PhD^1^, Stephan Zipfel MD^141^, Andrew W Bergen PhD^142,143,c^, Joseph M Boden PhD^144,c^, Harry Brandt MD^145,c^, Steven Crawford MD^145,c^, Katherine A Halmi MD^146,c^, L. John Horwood MSc^144,c^, Craig Johnson PhD^147,c^, Allan S Kaplan MSc, MD, FRCP(C)^85,c^, Walter Kaye MD^148,c^, James Mitchell MD^149,c^, Catherine M Olsen PhD, MPH^13,c^, John F Pearson PhD^150,c^, Nancy L Pedersen PhD^6,c^, Michael Strober PhD^151,152,c^, Thomas Werge PhD^153,c^, David C Whiteman MBBS(Hons), PhD, FAFPHM^13,c^, D. Blake Woodside MD^154,155,c^, Garret D Stuber PhD^1,156^, Scott Gordon PhD^13,d^, Jakob Grove PhD^9,157,158,159,d^, Anjali K Henders BSc(Hons)^160,d^, Anders Juréus PhD^6,d^, Katherine M Kirk PhD^13,d^, Janne T Larsen MSc^157,161,162,d^, Richard Parker BA(Hons)^13,d^, Liselotte Petersen PhD^157,161,162,d^, Jennifer Jordan PhD^163,164,e^, Martin Kennedy PhD^165,e^, Grant W Montgomery PhD^13,160,166,e^, Tracey D Wade PhD^167,e^, Andreas Birgegård PhD^10,11,e^, Paul Lichtenstein PhD^6,e^, Claes Norring PhD^10,11,e^, Mikael Landén MD, PhD^6,20,f^, Nicholas G Martin PhD^13,f^, Preben Bo Mortensen MD, DrMedSc^157,161,162,f^, Patrick F Sullivan MD, FRANZCP^1,4,6,f^, Gerome Breen PhD^5,7,f^, Cynthia M Bulik PhD^1,6,168,f,*^

a Co-second authors

b Secondary analysts/writing group

c Provided Anorexia Nervosa Genetics Initiative (ANGI) controls and extra samples

d ANGI contributors

e ANGI investigators

f ANGI principal investigators and PGC-ED co-chairs and co-senior authos

Affiliations:

1 Department of Psychiatry, University of North Carolina at Chapel Hill, Chapel Hill, North Carolina, US

2 School of Psychology, Curtin University, Perth, Australia

3 School of Paediatrics and Child Health, University of Western Australia, Perth, Australia

4 Department of Genetics, University of North Carolina at Chapel Hill, Chapel Hill, North Carolina, US

5 Institute of Psychiatry, Psychology and Neuroscience, Social, Genetic and Developmental Psychiatry (SGDP) Centre, King’s College London, London, UK

6 Department of Medical Epidemiology and Biostatistics, Karolinska Institutet, Stockholm, Sweden

7 National Institute for Health Research Biomedical Research Centre, King’s College London and South London and Maudsley National Health Service Trust, London, UK

8 Department of Child and Adolescent Psychiatry, University Hospital Essen, University of Duisburg-Essen, Essen, Germany

9 Department of Biomedicine, Aarhus University, Aarhus, Denmark

10 Department of Clinical Neuroscience, Karolinska Institutet, Stockholm, Sweden

11 Center for Psychiatry Research, Stockholm Health Care Services, Stockholm City Council, Stockholm, Sweden

12 Department of Psychiatry, Psychosomatics and Psychotherapy, University of Würzburg, Würzburg, Germany

13 QIMR Berghofer Medical Research Institute, Brisbane, Australia

14 Analytic and Translational Genetics Unit, Massachusetts General Hospital, Boston, Massachusetts, US

15 Stanley Center for Psychiatric Research, Broad Institute of the Massachusetts Institute of Technology and Harvard University, Cambridge, Massachusetts, US

16 Department of Psychiatry and Psychotherapy, Charité - Universitätsmedizin, Berlin, Germany

17 Department of Medical and Molecular Genetics, King’s College London, Guy’s Hospital, London, UK

18 Brain Center Rudolf Magnus, Department of Translational Neuroscience, University Medical Center Utrecht, Utrecht, The Netherlands

19 Center for Eating Disorders Rintveld, Altrecht Mental Health Institute, Zeist, The Netherlands

20 Sahlgrenska Academy, University of Gothenburg, Gothenburg, Sweden

21 Institute of Environmental Medicine, Karolinska Institutet, Stockholm, Sweden

22 Department of Behavioral Medicine, National Institute of Mental Health, National Center of Neurology and Psychiatry, Tokyo, Japan

23 NORMENT KG Jebsen Centre, Division of Mental Health and Addiction, University of Oslo, Oslo University Hospital, Oslo, Norway

24 Department of Psychiatry, Center for Neurobiology and Behavior, Perelman School of Medicine at the University of Pennsylvania, Philadelphia, Pennsylvania, US

25 Division of Psychological and Social Medicine and Developmental Neurosciences, Faculty of Medicine, Technische Universität Dresden, Dresden, Germany

26 INSERM U894, Centre of Psychiatry and Neuroscience, Paris, France

27 Wellcome Sanger Institute, Wellcome Genome Campus, Hinxton, Cambridge, UK

28 Department of Medical Biology, School of Medicine, University of Split, Split, Croatia

29 Department of Child and Adolescent Psychiatry, Psychosomatics and Psychotherapy, RWTH Aachen University, Aachen, Germany

30 Klinikum Frankfurt/Oder, Frankfurt, Germany

31 Clinical Genetics Unit, Department of Woman and Child Health, University of Padova, Padova, Italy

32 Institute of Medical Genetics and Pathology, University Hospital Basel, Basel, Switzerland

33 Life Sciences Institute and Department of Molecular and Integrative Physiology, University of Michigan, Ann Arbor, Michigan, US

34 Department of Emergency Psychiatry and Post-Acute Care, CHRU Montpellier, University of Montpellier, Montpellier, France

35 Department of Psychiatry, University of Minnesota, Minneapolis, Minnesota, US

36 MRC Integrative Epidemiology Unit, University of Bristol, Bristol, UK

37 School of Social and Community Medicine, University of Bristol, Bristol, UK

38 Department of Psychosomatic Medicine and Psychotherapy, Hannover Medical School, Hannover, Germany

39 Department of Nutrition and Dietetics, Harokopio University, Athens, Greece

40 Department of Neurosciences, University of Padova, Padova, Italy

41 College of Nursing, Seattle University, Seattle, Washington, US

42 Department of Psychology, Virginia Commonwealth University, Richmond, Virginia, US

43 Department of Psychiatry, Athens University Medical School, Athens University, Athens, Greece

44 L’institut du thorax, INSERM, CNRS, UNIV Nantes, Nantes, France

45 L’institut du thorax, CHU Nantes, Nantes, France

46 Department of Psychiatric Genetics, Poznan University of Medical Sciences, Poznan, Poland

47 Barcelona Institute of Science and Technology, Barcelona, Spain

48 Universitat Pompeu Fabra, Barcelona, Spain

49 Centro de Investigación Biomédica en Red en Epidemiología y Salud Pública (CIBERESP), Barcelona, Spain

50 Department of Psychiatry and Behavioral Sciences, Stanford University, Stanford, California, US

51 Department of Child and Adolescent Psychiatry, Psychosomatics and Psychotherapy, University Hospital of Würzburg, Centre for Mental Health, Würzburg, Germany

52 Estonian Genome Center, University of Tartu, Tartu, Estonia

53 Program in Medical and Population Genetics, Broad Institute of the Massachusetts Institute of Technology and Harvard University, Cambridge, Massachusetts, US

54 Genomics and Disease, Bioinformatics and Genomics Programme, Centre for Genomic Regulation, Barcelona, Spain

55 Department of Psychiatry, University Hospital of Bellvitge –IDIBELL and CIBERobn, Barcelona, Spain

56 Department of Clinical Sciences, School of Medicine, University of Barcelona, Barcelona, Spain

57 Department of Psychiatry and Psychotherapy, Ludwig-Maximilians-University (LMU), Munich, Germany

58 Schön Klinik Roseneck affiliated with the Medical Faculty of the University of Munich (LMU), Munich, Germany

59 Department of Cancer, Epidemiology and Genetics, Masaryk Memorial Cancer Institute, Brno, Czech Republic

60 Institute of Human Genetics, University of Bonn School of Medicine & University Hospital Bonn, Bonn, Germany

61 Department of Genomics, Life and Brain Center, University of Bonn, Bonn, Germany

62 Department of Psychiatry (UPK), University of Basel, Basel, Switzerland

63 Department of Biomedicine, University of Basel, Basel, Switzerland

64 Department of Surgery, Faculty of Medicine, University of Toronto, Toronto, Canada

65 Department of Psychiatry, Psychotherapy and Psychosomatics, Martin Luther University of Halle-Wittenberg, Halle, Germany

66 1st Psychiatric Department, National and Kapodistrian University of Athens, Medical School, Eginition Hospital, Athens, Greece

67 CMME (Groupe Hospitalier Sainte-Anne), Paris Descartes University, Paris, France

68 Center for Applied Genomics, Children’s Hospital of Philadelphia, Philadelphia, Pennsylvania, US

69 Department of Pediatrics, Perelman School of Medicine, University of Pennsylvania, Philadelphia, Pennsylvania, US

70 Department of Adult Psychiatry, Poznan University of Medical Sciences, Poznan, Poland

71 Zorg op Orde, Leidschendam, The Netherlands

72 Department of General Internal Medicine and Psychosomatics, Heidelberg University Hospital, Heidelberg University, Heidelberg, Germany

73 Department of Psychiatry, and Genetics and Genomics Sciences, Division of Psychiatric Genomics, Icahn School of Medicine at Mount Sinai, New York, New York, US

74 Biological Psychiatry Laboratory, McLean Hospital/Harvard Medical School, Boston, Massachusetts, US

75 Eating Disorders Unit, Parklandklinik, Bad Wildungen, Germany

76 Department of Molecular Life Science, Division of Basic Medical Science and Molecular Medicine, School of Medicine, Tokai University, Isehara, Japan

77 Department of Epidemiology and Public Health, University of Ostrava, University of Olomouc, Olomouc, Czech Republic

78 Rheumatology Research Group, Vall d’Hebron Research Institute, Barcelona, Spain

79 Department of Psychiatry, First Faculty of Medicine, Charles University, Prague, Czech Republic

80 Department of Public Health, University of Helsinki, Helsinki, Finland

81 Institute for Molecular Medicine Finland, Helsinki Institute of Life Science, University of Helsinki, Helsinki, Finland

82 Institute of Public Health and Clinical Nutrition, Department of Clinical Nutrition, University of Eastern Finland, Kuopio, Finland

83 Eating Disorders Unit, Department of Child and Adolescent Psychiatry, Medical University of Vienna, Vienna, Austria

84 Groningen Institute for Evolutionary Life Sciences, University of Groningen, Groningen, The Netherlands

85 Center for Addiction and Mental Health, Department of Psychiatry, Institute of Medical Science, University of Toronto, Toronto, Canada

86 Health Services Research Unit, University of Aberdeen, Aberdeen, UK

87 Department of Psychiatry, Seoul Paik Hospital, Inje University, Seoul, Korea

88 Rheumatology Unit, Department of Medicine, Center for Molecular Medicine, Karolinska Institutet and Karolinska University Hospital, Stockholm, Sweden

89 Department of Psychology, Michigan State University, East Lansing, Michigan, US

90 Department of Mental Disorders, Norwegian Institute of Public Health, Oslo, Norway

91 Department of Clinical Science, K.G. Jebsen Centre for Psychosis Research, Norwegian Centre for Mental Disorders Research (NORMENT), University of Bergen, Bergen, Norway

92 Dr. Einar Martens Research Group for Biological Psychiatry, Center for Medical Genetics and Molecular Medicine, Haukeland University Hospital, Bergen, Norway

93 Department of Clinical Medicine, Laboratory Building, Haukeland University Hospital, Bergen, Norway

94 Institute of Medical Science, University of Toronto, Toronto, Canada

95 American School of Professional Psychology, Argosy University, Northern Virginia, Arlington, Virginia, US

96 Department of Cancer Epidemiology and Prevention, M Skłodowska-Curie Cancer Center - Oncology Center, Warsaw, Poland

97 BESE Division, King Abdullah University of Science and Technology, Thuwal, Saudi Arabia

98 Department of Psychiatry, University of Lausanne-University Hospital of Lausanne (UNIL-CHUV), Lausanne, Switzerland

99 Department of Psychiatry, University of Campania "Luigi Vanvitelli", Naples, Italy

100 Center for Integrative Genomics, University of Lausanne, Lausanne, Switzerland

101 Department of Paediatric Laboratory Medicine, The Hospital for Sick Children, Toronto, Canada

102 Department of Psychiatry, University College Cork, Cork, Ireland

103 Eist Linn Adolescent Unit, Bessborough, Health Service Executive South, Cork, Ireland

104 Institute of Molecular and Cell Biology, University of Tartu, Tartu, Estonia

105 Molecular Epidemiology Section (Department of Medical Statistics), Leiden University Medical Centre, Leiden, The Netherlands

106 Department of Psychiatry, Faculty of Medicine, University of Geneva, Geneva, Switzerland

107 Division of Child and Adolescent Psychiatry, Geneva University Hospital, Geneva, Switzerland

108 National Center for PTSD, VA Boston Healthcare System, Department of Psychiatry, Boston University School of Medicine, Boston, Massachusetts, US

109 Department of Medicine, Surgery and Dentistry "Scuola Medica Salernitana", University of Salerno, Salerno, Italy

110 Department of Neuroscience, Psychology, Drug Research and Child Health (NEUROFARBA), University of Florence, Florence, Italy

111 Kartini Clinic, Portland, Oregon, US

112 Center for Neurobehavioral Genetics, Semel Institute for Neuroscience and Human Behavior, University of California Los Angeles, Los Angeles, California, US

113 Center for Human Genome Research at the Massachusetts General Hospital, Boston, Massachusetts, US

114 Saint Joan de Déu Research Institute, Saint Joan de Déu Barcelona Children’s Hospital, Barcelona, Spain

115 Institute of Biomedicine (IBUB), University of Barcelona, Barcelona, Spain

116 Department of Genetics, Microbiology and Statistics, University of Barcelona, Barcelona, Spain

117 Institute of Clinical Medicine, University of Oslo, Oslo, Norway

118 Department of Health Science, University of Florence, Florence, Italy

119 Department of Biometry, University of Helsinki, Helsinki, Finland

120 Eating Disorders Research and Treatment Center, Department of Child and Adolescent Psychiatry, Faculty of Medicine, Technische Universität Dresden, Dresden, Germany

121 Department of Psychiatry, Neurobiology, Pharmacology, and Biotechnologies, University of Pisa, Pisa, Italy

122 Department of Psychiatry, Poznan University of Medical Sciences, Poznan, Poland

123 Department of Neurosciences, Padua Neuroscience Center, University of Padova, Padova, Italy

124 Institute of Medical Statistics, Computer and Data Sciences, Jena University Hospital, Jena, Germany

125 Department of Genetics and Genomic Biology, The Hospital for Sick Children, Toronto, Canada

126 J. Craig Venter Institute (JCVI), La Jolla, California, US

127 Department of Psychiatry and Psychotherapy, Medical University of Vienna, Vienna, Austria

128 Department of Pediatrics and Center of Applied Genomics, First Faculty of Medicine, Charles University, Prague, Czech Republic

129 Center for Eating Disorders Ursula, Rivierduinen, Leiden, The Netherlands

130 Department of Psychiatry, Leiden University Medical Centre, Leiden, The Netherlands

131 Department of Child and Adolescent Psychiatry, Poznan University of Medical Sciences, Poznan, Poland

132 IRCSS Fondazione Don Gnocchi, Florence, Italy

133 Department of Environmental Epidemiology, Nofer Institute of Occupational Medicine, Lodz, Poland

134 Department of Psychiatry, University of Naples SUN, Naples, Italy

135 Department of Psychiatry, University of Perugia, Perugia, Italy

136 Brain Sciences Department, Stremble Ventures, Limassol, Cyprus

137 Adolescent Health Unit, Second Department of Pediatrics, "P. & A. Kyriakou" Children’s Hospital, University of Athens, Athens, Greece

138 Pediatric Intensive Care Unit, "P. & A. Kyriakou" Children’s Hospital, University of Athens, Athens, Greece

139 Faculty of Social and Behavioral Sciences, Utrecht University, Utrecht, The Netherlands

140 Institute of Translational Genomics, Helmholtz Zentrum München, Neuherberg, Germany

141 Department of Internal Medicine VI, Psychosomatic Medicine and Psychotherapy, University Medical Hospital Tuebingen, Tuebingen, Germany

142 BioRealm, LLC, Walnut, California, US

143 Oregon Research Institute, Eugene, Oregon, US

144 Christchurch Health and Development Study, University of Otago, Christchurch, New Zealand

145 The Center for Eating Disorders at Sheppard Pratt, Baltimore, Maryland, US

146 Department of Psychiatry, Weill Cornell Medical College, New York, New York, US

147 Eating Recovery Center, Denver, Colorado, US

148 Department of Psychiatry, University of California San Diego, San Diego, California, US

149 Department of Psychiatry and Behavioral Science, University of North Dakota School of Medicine and Health Sciences, Fargo, North Dakota, US

150 Biostatistics and Computational Biology Unit, University of Otago, Christchurch, New Zealand

151 Department of Psychiatry and Biobehavioral Science, Semel Institute for Neuroscience and Human Behavior, University of California Los Angeles, Los Angeles, California, US

152 David Geffen School of Medicine, University of California Los Angeles, Los Angeles, California, US

153 Department of Clinical Medicine, University of Copenhagen, Copenhagen, Denmark

154 Department of Psychiatry, Faculty of Medicine, University of Toronto, Toronto, Canada

155 Toronto General Hospital, Toronto, Canada

156 Department of Cell Biology and Physiology, University of North Carolina at Chapel Hill, Chapel Hill, North Carolina, US

157 The Lundbeck Foundation Initiative for Integrative Psychiatric Research (iPSYCH), Aarhus, Denmark

158 Centre for Integrative Sequencing, iSEQ, Aarhus University, Aarhus, Denmark

159 Bioinformatics Research Centre, Aarhus University, Aarhus, Denmark

160 Institute for Molecular Bioscience, University of Queensland, Brisbane, Australia

161 National Centre for Register-Based Research, Aarhus BSS, Aarhus University, Aarhus, Denmark

162 Centre for Integrated Register-based Research (CIRRAU), Aarhus University, Aarhus, Denmark

163 Department of Psychological Medicine, University of Otago, Christchurch, New Zealand

164 Canterbury District Health Board, Christchurch, New Zealand

165 Department of Pathology and Biomedical Science, University of Otago, Christchurch, New Zealand

166 Queensland Brain Institute, University of Queensland, Brisbane, Australia

167 School of Psychology, Flinders University, Adelaide, Australia

168 Department of Nutrition, University of North Carolina at Chapel Hill, Chapel Hill, North Carolina, US
