# Supplementary material for: Genomics of body fat percentage may contribute to sex bias in anorexia nervosa
Source: Am J Med Genet B Neuropsychiatr Genet. 2018 Dec 28;180(6):428–38. doi: 10.1002/ajmg.b.32709 (PMC6751355; doi:10.1002/ajmg.b.32709)
Supplement: Supplementary file 5 — Appendix S3 Supporting Information [file AJMG-180-428-s005.docx]

**MAGIC (the Meta-Analyses of Glucose and Insulin-related traits Consortium) investigators**

Vasiliki Lagou^1-3^, Reedik Mägi^4^, Jouke-Jan J Hottenga^5-6^, Harald Grallert^7^, John RB Perry^1,8^, Nabila Bouatia-Naji^9-11^, Letizia Marullo^12^, Denis Rybin^13^, Rick Janssen^14^, Josine L Min^15-16^, Antigone S Dimas^17-18^, Joao Fadista^19^, Maria Stathopoulou^20^, Aaron Isaacs^21-23^, Sara M Willems^24^, Pau Navarro^25^, Toshiko Tanaka^26^, Anne U Jackson^27^, May E Montasser^28^, Jeff R O'Connell^28^, Lawrence F Bielak^29^, Rebecca J Webster^30^, Richa Saxena^31-34^, Jeanette S Andrews^35^, Beate St Pourcain^36^, Nicholas J Timpson^37^, Perttu Salo^38^, So-Youn Shin^39^, Najaf Amin^40^, Albert V Smith^41-43^, Guo Li^44-45^, Niek Verweij^46^, Anuj Goel^1^, Ian Ford^47^, Paul CD Johnson^47-48^, Toby Johnson^49-50^, Karen Kapur^51^, Gudmar Thorleifsson^52^, Rona Strawbridge^53-54^, Laura J Rasmussen-Torvik^55^, Tõnu Esko^4,56^, Evelin Mihailov^56^, Tove Fall^57^, Andrea Groop^58-60^, Fraser Ross^61^, Anubha Mahajan^62^, Stavroula Kanoni^63^, Vilmantas Giedraitis^64^, Marcus E Kleber^65-66^, Günther Silbernagel^67^, Julia Meyer^68^, Martina Müller-Nurasyid^69-71^, Andrea Ganna^58-60^, Antti-Pekka Sarin^72-73^, Loic Yengo^9-10^, Dmitry Shungin^74-76^, Jian'an Luan^77^, Momoko Horikoshi^1,78-79^, An Ping^80^, Sanna Serena^81-82,82^, Yvonne Boettcher^83-84^, N W Rayner^1,63,78^, Ilja M Nolte^85^, Tatijana Zemunik^86^, Erik van Iperen^87^, Peter Kovacs^88^, Nicholas D Hastie^25^, Sarah H Wild^61^, Stela McLachlan^61^, Susan Campbell^25^, Ozren Polasek^86^, Olga Carlson^89^, Josephine Egan^89^, Wieland Kiess^84,90^, Gonneke Willemsen^5^, Johanna Kuusisto^91^, Markku Laakso^91^, Maria Dimitriou^92^, Andrew A Hicks^93^, Rainer Rauramaa^94-95^, Stefania Bandinelli^96^, Barbara Thorand^97^, Yongmei Liu^98^, Iva Miljkovic^99^, Lars Lind^100^, Alex Doney^101^, Markus Perola^4,38,102^, Aroon Hingorani^103^, Mika Kivimaki^103^, Meena Kumari^103-104^, Amanda J Bennett^78^, Christopher J Groves^78^, Mark I McCarthy^1,78,105^, Christian Herder^106-107^, Ulf de Faire^108^, Stephan JL Bakker^109^, Matti Uusitupa^110^, Colin NA Palmer ^101^, J W Jukema^111^, Naveed Sattar^112^, Anneli Pouta ^113-114^, Harold Snieder^85,115^, Eric Boerwinkle^116-117^, James S Pankow^118^, Patrik K Magnusson^119^, Ulrika Krus^120^, Eco JCN de Geus^5-6^, Matthias Blüher^83-84^, Bruce HR Wolffenbuttel^115,121^, Michael A Province^80^, Goncalo R Abecasis^82,122^, James B Meigs^123-124^, Kees G Hovingh^125^, Jaana Lindström^126^, James F Wilson^61,127^, Alan F Wright^127^, George V Dedousis^92^, Stefan R Bornstein^128^, Peter EH Schwarz^128^, Anke Tönjes^83-84^, Bernhard R Winkelmann^129^, Bernhard O Boehm^130^, Winfried März^66,131^, Andres Metspalu^4,56^, Jackie F Price^61^, Panos Deloukas^63,132-133^, Antje Körner^84,90^, Timo A Lakka^94,134^, Sirkka M Keinanen-Kiukaanniemi^135-136^, Timo E Saaristo^137-138^, Richard N Bergman^139^, Jaakko Tuomilehto^140-143^, Nicholas J Wareham^77^, Claudia Langenberg^77^, Satu Männistö^144^, Paul W Franks^75,145-146^, Caroline Hayward^25^, Veronique Vitart^25^, Jaako Kaprio^147^, Sophie Visvikis-Siest^20^, Beverley Balkau^148-149^, David Altshuler^31-32,124^, Igor Rudan^61^, Michael Stumvoll^83-84^, Harry Campbell^61^, Cornelia M van Duijn^21,150^, Christian Gieger^151-153^, Thomas Illig^151,154-155^, Luigi Ferrucci^26^, Nancy L Pedersen^119^, Peter P Pramstaller^93,156-157^, Michael Boehnke^27^, Timothy M Frayling^8^, Alan R Shuldiner^28,158^, Patricia A Peyser^29^, Lyle J Palmer^159^, Brenda W Penninx^160^, Pierre Meneton^161^, Tamara B Harris^162^, Gerjan Navis^109^, Pim van der Harst^46,163^, George Davey Smith^37^, Nita G Forouhi^77^, Ruth JF Loos^77,164^, Veikko Salomaa^165^, Nicole Soranzo^39^, Dorret I Boomsma^5^, Beverley Balkau^148-149^, Leif Groop^166-168^, Tiinamaija Tuomi^169-171^, Albert Hofman^40,172^, Patricia B Munroe^49-50^, Vilmundur Gudnason^41,173^, David S Siscovick^44-45,174^, Hugh Watkins^1^, Cecile Lecoeur^9-10^, Peter Vollenweider^175^, Kari Stefansson^52,176^, Marjo-Riitta Jarvelin ^177-178^, Anders Hamsten^53-54,179^, George Nicholson^180^, Fredrik Karpe^78,105^, Emmanouil T Dermitzakis^18^, Cecilia M Lindgren^1,78,181^, Philippe Froguel^9-10,182^, Valeriya Lyssenko^168,183^, Richard M Watanabe^184-186^, Erik Ingelsson^57,187^, Jose C Florez^32,188^, Josée Dupuis^189-190^, Inês Barroso^191-192^, Andrew P Morris^1,4,193^, Inga Prokopenko^1,78,182^.

**Affiliations:**

1)Wellcome Centre for Human genetics, University of Oxford, Oxford, United Kingdom; 2)Laboratory for Neuroimmunology, Department of Neurosciences, KU Leuven, Leuven, Belgium; VIB Center for Brain & Disease Research, Leuven, Belgium; 3)Laboratory for Translational Immunology, Department of Immunology and Microbiology, KU Leuven, Leuven, Belgium; 4)Estonian Genome Center, University of Tartu, Tartu, Estonia; 5)Department of Biological Psychology, Vrije Universiteit, Amsterdam, the Netherlands; 6)Amsterdam Public Health research institute, VU University medical center, Amsterdam, the Netherlands; 7)Research Unit Molecular Epidemiology, Helmholtz Zentrum München, German Research Center for Environmental Health, Neuherberg, Germany; 8)Genetics of Complex Traits, Peninsula Medical School, University of Exeter, United Kingdom; 9)University of Lille Nord de France, Lille, France; 10)CNRS UMR8199, Institut Pasteur de Lille, Lille, France; 11)INSERM U970, Paris Cardiovascular Research Center PARCC, 75006 Paris, France; 12)Department of Life Sciences and Biotechnology, University of Ferrara, Ferrara, Italy; 13)Boston University Data Coordinating Center, Boston, Massachusetts, USA; 14)Department of Psychiatry, VU University Medical Center Amsterdam, Amsterdam, the Netherlands; 15)MRC Integrative Epidemiology Unit, University of Bristol, Bristol, United Kingdom; 16)Bristol Medical School, University of Bristol, Bristol, United Kingdom; 17)Biomedical Sciences Research Center "Alexander Fleming", Vari, Greece; 18)Department of Genetic Medicine and Development, University of Geneva Medical School, Geneva, Switzerland; 19)Department of Epidemiology Research, Statens Serum Institut, Copenhagen, Denmark; 20)UMR INSERM U1122; Interactions Gène-Environnement en Physiopathologie Cardio-Vasculaire (IGE-PCV), Université de Lorraine, Nancy, France; 21)Genetic Epidemiology Unit, Department of Epidemiology, Erasmus Medical Center, Rotterdam, the Netherlands; 22)CARIM School for Cardiovascular Diseases, Maastricht Centre for Systems Biology (MaCSBio), Maastricht University, Maastricht, the Netherlands; 23)Department of Biochemistry, Maastricht University, Maastricht, the Netherlands; 24)Genetic Epidemiology Unit, Department of Epidemiology, Erasmus University Medical Center, Rotterdam, the Netherlands; 25)MRC Human Genetics Unit, MRC Institute of Genetics and Molecular Medicine,University of Edinburgh, Western General Hospital, Edinburgh, United Kingdom; 26)Clinical Research Branch, National Institute on Aging, Baltimore, Maryland, USA; 27)Department of Biostatistics and Center for Statistical Genetics, University of Michigan, Ann Arbor, Michigan, USA; 28)Division of Endocrinology, Diabetes, and Nutrition, Department of Medicine, University of Maryland, School of Medicine, Baltimore, Maryland, USA; 29)Department of Epidemiology, University of Michigan, Ann Arbor, Michigan, USA; 30)Laboratory for Cancer Medicine, Harry Perkins Institute of Medical Research, University of Western Australia Centre for Medical Research, Nedlands, Australia; 31)Broad Institute of Harvard and Massachusetts Institute of Technology (MIT), Cambridge, Massachusetts, USA; 32)Center for Human Genetic Research, Massachusetts General Hospital, Boston, Massachusetts, USA; 33)Department of Genetics, Harvard Medical School, Boston, Massachusetts, USA; 34)Departmentartment of Anesthesia, Critical Care and Pain Medicine, MGH, Boston, USA; 35)Department of Biostatistical Sciences, Division of Public Health Sciences, Wake Forest University School of Medicine, Winston-Salem, North Carolina, USA; 36)School of Social and Community Medicine, University of Bristol, Bristol, United Kingdom; 37)MRC CAiTE Centre, School of Social and Community Medicine, University of Bristol, Bristol, United Kingdom; 38)Public Health Genomics Unit, Department of Chronic Disease Prevention, the National Institute for Health and Welfare, Helsinki, Finland; 39)Wellcome Trust Sanger Institute, Wellcome Trust Genome Campus, Hinxton, United Kingdom; 40)Department of Epidemiology Erasmus MC, Rotterdam, the Netherlands; 41)Icelandic Heart Association, Kopavogur, Iceland; 42)Faculty of Medicine, University of Iceland, Reykjavik, Iceland; 43)Department of Biostatistics, School of Public Health,University of Michigan, Ann Arbor, Michigan, USA; 44)Cardiovascular Health Research Unit, University of Washington, Seattle, Washington, USA; 45)Department of Medicine, University of Washington, Seattle, Washington, USA; 46)Department of Cardiology, University Medical Center Groningen, University of Groningen, Groningen, the Netherlands; 47)Robertson Centre for Biostatistics, University of Glasgow, Glasgow, United Kingdom; 48)Institute of Biodiversity, Animal Health & Comparative Medicine, University of Glasgow, Glasgow, United Kingdom; 49)Clinical Pharmacology, William Harvey Research Institute, Barts and The London School of Medicine and Dentistry, Queen Mary University of London, London, United Kingdom; 50)NIHR Barts Cardiovascular Biomedical Research Unit, Barts and The London School of Medicine and Dentistry, Queen Mary University of London, London, United Kingdom; 51)Department of Medical Genetics, University of Lausanne, Lausanne, Switzerland; 52)deCODE Genetics, Reykjavik, Iceland; 53)Cardiovascular Medicine Unit, Department of Medicine, Solna, Karolinska Institutet, Stockholm, Sweden; 54)Center for Molecular Medicine, Karolinska University Hospital Solna, Stockholm, Sweden; 55)Department of Preventive Medicine, Northwestern University Feinberg School of Medicine, Chicago, Illinois, USA; 56)Institute of Molecular and Cell Biology, University of Tartu, Tartu, Estonia; 57)Department of Medical Sciences, Molecular Epidemiology and Science for Life Laboratory, Uppsala University, Uppsala, Sweden; 58)Analytic and Translational Genetics Unit, Massachusetts General Hospital, Boston, Massachusetts, USA; 59)Program in Medical and Population Genetics, Broad Institute of MIT and Harvard, Cambridge, Massachusetts, USA; 60)Stanley Center for Psychiatric Research, Broad Institute of MIT and Harvard, Cambridge, Massachusetts, USA; 61)Usher Institute of Population Health Sciences and Informatics, University of Edinburgh, Edinburgh, United Kingdom; 62)Wellcome Centre for Human Genetics, University of Oxford, Oxford, United Kingdom; 63)Wellcome Trust Sanger Institute, Hinxton, United Kingdom; 64)Department of Public Health and Caring Sciences, Uppsala Universitet, Uppsala, Sweden; 65)LURIC Study nonprofit LLC, Freiburg, Germany; 66)Mannheim Institute of Public Health, Social and Preventive Medicine, Medical Faculty of Mannheim, University of Heidelberg, Mannheim, Germany; 67)Division of Angiology, Department of Internal Medicine, Medical University of Graz, Austria; 68)Institute of Genetic Epidemiology,

Helmholtz Zentrum München, German Research Center for Environmental Health, Neuherberg, Germany; 69)Institute of Medical Informatics, Biometry and Epidemiology, Chair of Epidemiology and Chair of Genetic Epidemiology, Ludwig-Maximilians-Universität, Munich, Germany; 70)Department of Medicine I, University Hospital Grosshadern, Ludwig-Maximilians-University, Munich, Germany; 71)Institute of Genetic Epidemiology, Helmholtz Zentrum München, German Research Center for Environmental Health, Neuherberg, Germany; 72)Institute for Molecular Medicine Finland, FIMM, University of Helsinki, Finland; 73)Public Health Genomics Unit, National Institute for Health and Welfare, Helsinki, Finland; 74)Department of Public Health & Clinical Medicine, Umeå University, Umeå, Sweden; 75)Department of Clinical Sciences, Genetic and Molecular Epidemiology Unit, Skåne University Hospital Malmö, Malmö, Sweden; 76)Department of Odontology, Umeå University, Umeå, Sweden; 77)MRC Epidemiology Unit, University of Cambridge School of Clinical Medicine, Cambridge, United Kingdom; 78)Oxford Centre for Diabetes, Endocrinology and Metabolism, University of Oxford, Oxford, United Kingdom; 79)RIKEN, Center for Integrative Medical Sciences, Laboratory for Endocrinology, Metabolism and kidney disease, Yokohama, Japan; 80)Division of Statistical Genomics, Washington University School of Medicine, St. Louis, Missouri, USA; 81)Istituto di Ricerca Genetica e Biomedica, CNR, Monserrato, Italy; 82) ; 83)University of Leipzig, Department of Medicine, Leipzig, Germany; 84)University of Leipzig, IFB Adiposity Diseases, Leipzig, Germany; 85)Department of Epidemiology, University Medical Center Groningen, University of Groningen, Groningen, the Netherlands; 86)Faculty of Medicine, University of Split, Split, Croatia; 87)Department of Clinical Epidemiology and Biostatistics, Academic Medical Center, University of Amsterdam, Amsterdam, the Netherlands; 88)Interdisciplinary Center for Clinical Research, University of Leipzig, Leipzig, Germany; 89)Laboratory of Clinical Investigation, National Institute of Aging, Baltimore, Maryland, USA; 90)Pediatric Research Center, Department of Women's & Child Health, University of Leipzig, Leipzig, Germany; 91)Department of Medicine, University of Eastern Finland and Kuopio University Hospital, Kuopio, Finland; 92)Department of Dietetics-Nutrition, Harokopio University, Athens, Greece; 93)Center for Biomedicine, European Academy Bozen/Bolzano (EURAC), Bolzano, Italy - Affiliated Institute of the University of Lübeck, Lübeck, Germany; 94)Kuopio Research Institute of Exercise Medicine, Kuopio, Finland; 95)Department of Clinical Physiology and Nuclear Medicine, Kuopio University Hospital, Kuopio, Finland; 96)Geriatric Unit, Azienda Sanitaria Firenze (ASF), Florence, Italy; 97)Institute of Epidemiology II, Helmholtz Zentrum München, German Research Center for Environmental Health, Neuherberg, Germany; 98)Department of Epidemiology and Prevention, Division of Public Health Sciences, Wake Forest University School of Medicine, Winston-Salem, North Carolina, USA; 99)Department of Epidemiology, Center for Aging and Population Health, University of Pittsburgh, Pittsburgh, Pennsylvania, USA; 100)Department of Medical Sciences, Uppsala University, Akademiska sjukhuset, Uppsala, Sweden; 101)Pat McPherson Centre for Pharmacogenetics and Pharmacogenomics, Division of Molecular and Clinical Medicine, Ninewells Hospital and Medical School, University of Dundee, Dundee, United Kingdom; 102)Institute for Molecular Medicine Finland FIMM, University of Helsinki, Finland; 103)Department of Epidemiology and Public Health, University College London, London, United Kingdom; 104)University of Essex, Wivenhoe Park, Colchester, Essex, United Kingdom; 105)Oxford National Institute for Health Research Biomedical Research Centre, Churchill Hospital, Oxford, United Kingdom; 106)Institute for Clinical Diabetology, German Diabetes Center, Leibniz Center for Diabetes Research, Heinrich Heine University Düsseldorf, Düsseldorf, Germany; 107)German Center for Diabetes Research (DZD), München-Neuherberg, Germany; 108)Division of Cardiovascular Epidemiology, Institute of Environmental Medicine, Karolinska Institutet, Stockholm, Sweden; 109)Department of Internal Medicine, University Medical Center Groningen, University of Groningen, the Netherlands; 110)Institute of Public Health and Clinical Nutrition, University of Eastern Finland, Kuopio, Finland; 111)Department of Cardiology C5-P, Leiden University Medical Center, Leiden, the Netherlands; 112)Institute of Cardiovascular and Medical Sciences, University of Glasgow, Glasgow, United Kingdom; 113)National Institute for Health and Welfare, Oulu, Finland; 114)Department of Clinical Sciences/Obstetrics and Gynecology, University of Oulu, Oulu, Finland; 115)Lifelines Cohort Study and Biobank, Groningen, the Netherlands; 116)IMM Center for Human Genetics, University of Texas Health Science Center at Houston, Houston, Texas, USA; 117)Division of Epidemiology, School of Public Health, University of Texas Health Science Center at Houston, Houston, Texas, USA; 118)Division of Epidemiology and Community Health, School of Public Health, University of Minnesota, Minneapolis, Minnesota, USA; 119)Department of Medical Epidemiology and Biostatistics, Karolinska Institutet, Stockholm, Sweden; 120)Department of Clinical Sciences, Diabetes and Endocrinology Research Unit, University Hospital Malmö, Lund University, Malmö, Sweden ; 121)Department of Endocrinology, University Medical Center Groningen, University of Groningen, Groningen, the Netherlands; 122)Center for Statistical Genetics, Department of Biostatistics, University of Michigan, Ann Arbor, Michigan, USA; 123)General Medicine Division, Massachusetts General Hospital, Boston, Massachusetts, USA; 124)Department of Medicine, Harvard Medical School, Boston, Massachusetts, USA; 125)Department Vascular Medicine, Academic Medical Center, Amsterdam, the Netherlands; 126)National Institute for Health and Welfare, Diabetes Prevention Unit, Helsinki, Finland; 127)MRC Human Genetics Unit, MRC Institute of Genetics and Molecular Medicine, University of Edinburgh, Western General Hospital, Edinburgh, United Kingdom; 128)Department of Medicine III, University of Dresden, Medical Faculty Carl Gustav Carus, Dresden, Germany; 129)Cardiology Group, Frankfurt-Sachsenhausen, Germany; 130)Division of Endocrinology and Diabetes, Department of Medicine, University Hospital, Ulm, Germany; 131)Synlab Academy, Mannheim, Germany; 132)William Harvey Research Institute, Barts and The London School of Medicine and Dentistry, Queen Mary University of London, London, United Kingdom; 133)Princess Al-Jawhara Al-Brahim Centre of Excellence in Research of Hereditary Disorders (PACER-HD), King Abdulaziz University, Jeddah, Saudi Arabia; 134)Institute of Biomedicine/Physiology, University of Eastern Finland, Kuopio Campus, Kuopio, Finland; 135)Faculty of Medicine, Institute of Health Sciences, University of Oulu, Oulu, Finland; 136)Unit of General Practice, Oulu University Hospital, Oulu, Finland; 137)Finnish Diabetes Association, Tampere, Finland; 138)Pirkanmaa Hospital District, Tampere, Finland; 139)Diabetes and Obesity Research Institute, Cedars-Sinai Medical Center, Los Angeles, California, USA; 140)Department of Chronic Disease Prevention, National Institute for Health and Welfare, Helsinki, Finland; 141)Dasman Diabetes Institute, Dasman, Kuwait; 142)Centre for Vascular Prevention, Danube-University Krems, Krems, Austria; 143)Diabetes Research Group, King Abdulaziz University, Jeddah, Saudi Arabia; 144)Department of Public Health Solutions, National Institute for Health and Welfare, Helsinki, Finland; 145)Department of Nutrition, Harvard School of Public Health, Boston, Massachusetts, USA; 146)Department of Public Health & Clinical Medicine, Units of Medicine and Nutritional Research, Umeå University, Umeå, Sweden; 147)Department of Public Health, University of Helsinki, Helsinki, Finland; 148)Inserm, CESP Center for Research in Epidemiology and Public Health, U1018, Epidemiology of diabetes, obesity and chronic kidney disease over the lifecourse, Villejuif, France; 149)University Paris Sud 11, UMRS 1018, Villejuif, France; 150)Centre for Medical Systems Biology, Leiden, the Netherlands; 151)Research Unit of Molecular Epidemiology, Helmholtz Zentrum München, German Research Center for Environmental Health, Neuherberg, Germany; 152)Institute of Epidemiology II, Helmholtz Zentrum München, German Research Center for Environmental Health, Neuherberg, Germany; 153)German Center for Diabetes Research (DZD), Neuherberg, Germany; 154)Hannover Unified Biobank, Hannover Medical School, Hannover, Germany; 155)Institute of Human Genetics, Hannover Medical School, Hannover, Germany; 156)Department of Neurology, General Central Hospital, Bolzano, Italy; 157)Department of Neurology, University of Lübeck, Lübeck, Germany; 158)Geriatric Research and Education Clinical Center, Veterans Administration Medical Center, Baltimore, Maryland; 159)School of Public Health, University of Adelaide, Adelaide, Australia; 160)Department of Psychiatry, VU University Medical Center, Amsterdam, the Netherlands; 161)U872 Institut National de la Santé et de la Recherche Médicale, Centre de Recherche des Cordeliers, 75006 75006 Paris, France; 162)Geriatric Epidemiology Section, Laboratory of Epidemiology, Demography, and Biometry, National Institute on Aging, Bethesda, Maryland; 163)Department of Genetics, University Medical Center Groningen, University of Groningen, Groningen, the Netherlands; 164)The Charles Bronfman Institute for Personalized Medicine, Icahn School of Medicine at Mount Sinai, New York, NY, USA; 165)Chronic Disease Epidemiology and Prevention Unit, Department of Chronic Disease Prevention, National Institute for Health and Welfare, Helsinki, Finland; 166)Institute for Molecular Medicine, Helsinki, Finland ; 167)Department of Medicine, Helsinki University Hospital, University of Helsinki, Helsinki, Finland; 168)Department of Clinical Sciences, Diabetes and Endocrinology Research Unit, University Hospital Malmö, Lund University, Malmö, Sweden; 169)Endocrinology, Abdominal Centre, University of Helsinki and Helsinki University Hospital, Helsinki, Finland; 170)Diabetes and Obesity Research Program, University of Helsinki and Folkhälsan Research Center, Helsinki, Finland; 171)Finnish Institute for Molecular Medicine, University of Helsinki, Helsinki, Finland; 172)Netherlands Consortium for healthy ageing, the Hague, the Netherlands; 173)Faculty of Medicien University of Iceland, Reykjavik, Iceland; 174)Department of Epidemiology, University of Washington, Seattle, Washington, USA; 175)Department of Medicine, University Hospital Lausanne, Lausanne, Switzerland; 176)Faculty of Medicine, University of Iceland, Reykjavík, Iceland; 177)Department of Epidemiology and Biostatistics and HPA-MRC Center, School of Public Health, Imperial College London, London, United Kingdom; 178)Institute of Health Sciences, University of Oulu, Finland; 179)Department of Cardiology, Karolinska University Hospital Solna, Stockholm, Sweden; 180)Department of Statistics, University of Oxford, Oxford, United Kingdom; 181)Big Data Institute, Li Ka Shing Centre for Health Information and Discovery, University of Oxford, Oxford, United Kingdom; 182)Department of Medicine, Imperial College London, London, United Kingdom; 183)Department of Clinical Science, University of Bergen, Bergen, Norway; 184)Department of Preventive Medicine, Keck School of Medicine of USC, Los Angeles, California, USA; 185)Department of Physiology & Neuroscience, Keck School of Medicine of USC, Los Angeles, California, USA; 186)USC Diabetes and Obesity Research Institute, Los Angeles, California, USA; 187)Department of Medicine, Division of Cardiovascular Medicine, Stanford University School of Medicine, Stanford, California, USA; 188)Diabetes Research Center, Diabetes Unit, Massachusetts General Hospital, Boston, Massachusetts, USA; 189)Department of Biostatistics, Boston University School of Public Health, Boston, Massachusetts, USA; 190)National Heart, Lung, and Blood Institute's Framingham Heart Study, Framingham, Massachusetts, USA; 191)Wellcome Trust Sanger Institute, Wellcome Trust Genome Campus,Hinxton, United Kingdom; 192)University of Cambridge Metabolic Research Laboratories and NIHR Cambridge Biomedical Research Centre, Wellcome Trust-MRC Institute of Metabolic Science, Cambridge, United Kingdom; 193)Department of Biostatistics, University of Liverpool, Liverpool, United Kingdom;
